# DNA-Aeon provides flexible arithmetic coding for constraint adherence and error correction in DNA storage

**DOI:** 10.1038/s41467-023-36297-3

**Published:** 2023-02-06

**Authors:** Marius Welzel, Peter Michael Schwarz, Hannah F. Löchel, Tolganay Kabdullayeva, Sandra Clemens, Anke Becker, Bernd Freisleben, Dominik Heider

**Affiliations:** 1 Department of Mathematics and Computer Science, University of Marburg, Marburg, Germany; 2 Center for Synthetic Microbiology (SYNMIKRO), University of Marburg, Marburg, Germany

**Keywords:** Computational biology and bioinformatics, Computational platforms and environments, Software, DNA computing and cryptography

## Abstract

The extensive information capacity of DNA, coupled with decreasing costs for DNA synthesis and sequencing, makes DNA an attractive alternative to traditional data storage. The processes of writing, storing, and reading DNA exhibit specific error profiles and constraints DNA sequences have to adhere to. We present DNA-Aeon, a concatenated coding scheme for DNA data storage. It supports the generation of variable-sized encoded sequences with a user-defined Guanine-Cytosine (GC) content, homopolymer length limitation, and the avoidance of undesired motifs. It further enables users to provide custom codebooks adhering to further constraints. DNA-Aeon can correct substitution errors, insertions, deletions, and the loss of whole DNA strands. Comparisons with other codes show better error-correction capabilities of DNA-Aeon at similar redundancy levels with decreased DNA synthesis costs. In-vitro tests indicate high reliability of DNA-Aeon even in the case of skewed sequencing read distributions and high read-dropout.

## Abstract (web summary)

The extensive information capacity of DNA makes it an attractive alternative to traditional data storage. DNA-Aeon is a DNA data storage solution that can correct all error types commonly observed in DNA storage, while encoding data into sequences that meet user-defined constraints such as GC content, homopolymer length, and no undesired motifs.

## Introduction

The high rate of global digitization fosters an increasing demand for large-capacity data storage solutions. Conventional storage media either have a limited maximum information density (around $1{0}^{3}\,\frac{GB}{m{m}^{3}}$ for hard disc drives^1^) or have to be regularly replaced due to their short life expectancy^2,3^. DNA as a data storage medium is a promising alternative to traditional storage media for long-term data storage, thanks to its high information density and long life expectancy^4^. During the last years, tremendous progress has been made in the field of DNA data storage research^2,5–7^. To store digital data in DNA, it first has to be prepared in silico: the binary information is mapped to the four DNA nucleotides (nt) Adenine (A), Guanine (G), Cytosine (C), and Thymine (T). To increase the probability of successful data decoding in the presence of errors, additional redundancy is introduced in the form of an error-correcting code (ECC). Afterwards, the encoded data can be synthesized using various methods, most of which generate small fragments (oligonucleotides; short: oligos) of a length of 40–100 base pairs (bp)^8^. The synthesized fragments are then commonly stored in vitro. In vivo storage is a potential alternative, since it would allow the exploitation of a cell’s internal DNA repair systems for preventing the occurrence of errors during storage^1^. To read DNA fragments, sequencing technologies are used. They generate text files that contain the order of the different nucleotides of the DNA strand read by the sequencer, together with information regarding the uncertainty of the sequencer regarding the nucleotides, i.e., the quality of the base calls.

Each of these methods has characteristic error profiles and different constraints a DNA sequence has to adhere to^8,9^. Typical constraints include a Guanine-Cytosine (GC) content between 40 and 60% in short intervals and no homopolymers (repetitive stretches of the same nucleotide) longer than 3 or 4 nt. Another often overlooked constraint^2^ are undesired motifs, which could be restriction sites used for the DNA synthesis process, motifs with biological relevance, or motifs that increase the probability of sequencing errors^8^. If such motifs occur in the encoded DNA, they could lead to fragments that are not synthesizable, PCR amplification with reduced yield, or highly erroneous sequencing data. Löchel et al.^2^ developed a fractal-based method called mCGR, that is derived from chaos game representation to generate codewords that adhere to user-defined constraints, namely GC content, homopolymers, and undesired motifs. The codebooks generated using this method are one way to adhere to constraints.

In recent years, tremendous progress has been achieved in the field of DNA data storage systems, e.g., codes that combine error correction and constraint adherence. Most codes that are available as open-source software implementations follow a concatenated coding scheme, allowing to exploit the strengths of two or more codes while mitigating the weaknesses of a single code. For example, Grass et al.^10^ used a concatenation of two Reed-Solomon (RS) codes to correct individual base substitutions and also erasures of entire sequences. The digital data is mapped to elements of the Galois field *GF*(47), where each element of the field is represented by a DNA triplet that has different bases on the second and third positions, thereby avoiding the formation of homopolymers longer than three bases.

Erlich and Zielinski^11^ used fountain codes for storing data in DNA by treating the synthesized DNA fragments as packets in a data stream. An inner RS code protects each fragment, which allows the correction of some substitutions. If the RS code detects errors that it cannot correct (e.g., insertion and deletion errors (indels) or too many substitutions), the individual fragment is treated as an erasure. Erasures can be reconstructed from the other fragments by the outer Luby-Transform fountain code. Since fountain codes can generate a large number of packets from an input file, the authors added a constraint evaluation function to their software. A screening method discards all packets that do not adhere to user-defined homopolymer lengths and GC content constraints. The generation of new packets progresses until a predefined number of constraint-adhering packets is reached.

Press et al.^12^ used a hash-based convolutional code as an inner code that can correct indels directly, i.e., without treating a complete fragment as an erasure, as well as substitutions. An outer RS block code reconstructs fragments that are too damaged for the inner code to correct. The available software supports user-defined homopolymer lengths and GC contents by reducing the number of choices the encoder has, depending on the previously encoded bases.

Several other works in the literature provide solutions for challenges in the field of DNA data storage, e.g., image processing for DNA storage^13^, adaptation of the JPEG image coding algorithm for DNA data storage^14^, error correction codes using LDPC^15^ or Polar codes^16^, random access solutions^6,17,18^, and constrained codes^19^.

We present a method derived from arithmetic codes to encode binary data into constraint-adhering DNA sequences using codebooks. Furthermore, we exploit the redundancy introduced into the sequences for constraint adherence to correct insertions, deletions, and substitutions using a sequential decoding algorithm. Finally, we concatenate our code with NOREC4DNA^7^, a Raptor fountain code^20^ implementation, using the quality information of the sequential decoding process as an additional input. The fountain code uses this quality information to choose the packets used for the decoding process.

## Results

### Overview of the codes evaluated in this work

To evaluate DNA-Aeon, we compared it to three published codes with open-source implementations: the code published by Grass et al.^10^ (further referred to as Grass code), DNA Fountain^11^, and HEDGES^12^ (we will further refer to Hedges as the complete construct of inner-outer concatenated code that the authors described, and HEDGES for the inner code). The general features of each code implementation are shown in Table 1. The Grass code has a fixed block size of 713 strands of 118 bases each. Given the nature of fountain codes, DNA Fountain does not have a fixed block or strand size. However, in our evaluations, it required a sizeable minimal amount of bases to be able to reconstruct the input data, even in the absence of errors. Hedges has a fixed block size of 255 strands. The strand length of Hedges is somewhat variable, since it depends on the length of the used primers and the size of the input data. The largest coding strand length of Hedges is 254 bases. For DNA-Aeon, both the strand length and the number of fragments are freely selectable, with a minimal requirement of the input file size, plus a small overhead of the Raptor fountain code and four bases per packet for the required final CRC of each strand. If a header chunk is used, the minimal amount of bases per packet increases by the number of bases required to store the filename in the header chunk. While there is no maximum strand length, short to moderate strand lengths are recommended, as the loss of multiple smaller strands is easier compensated than the loss of one long strand by the outer code of DNA-Aeon. The DNA Fountain, Hedges, and DNA-Aeon implementations can adhere to the common constraints of homopolymer length and GC content. However, DNA-Aeon further supports motif constraints with the supplied codebook generation tool and other types of constraints by user-provided codebooks. The Grass code averts the formation of homopolymers of length 3 in the encoded data. All codes can correct substitutions and erasures of some strands, with Hedges, DNA Fountain, and DNA-Aeon being also able to correct indel errors. DNA-Aeon further encodes not only the file contents but also the metadata, such as file name, permissions, and file extension.

**Table 1:** Feature overview of the code implementations evaluated in our work

	Grass code	DNA Fountain	Hedges	DNA-Aeon
Scheme	Inner RS, outer RS	Inner RS, outer fountain (Luby transform)	Inner hash based, outer RS	Inner AC based, outer fountain (Raptor)
Block Size	713 strands, 118 bases per strand	Variable, high minimal requirement (100,000+ bases)	255 strands, semi-variable strand length	Variable
Constraints	HP 3	HP, GC	HP, GC	HP, GC, Motifs...
Error types	Substitutions, strand erasures	Substitutions, strand erasures, indels	Substitutions, strand erasures, indels	Substitution, strand erasures, indels

### Error correction performance

**Fig. 1:**
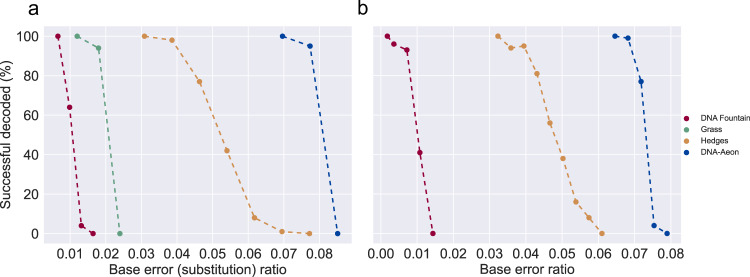
Base error ratio comparison: percentage of successful decoding attempts for a given base error (substitution) ratio (a) and percentage of successful decoding attempts for a given base error ratio (b), using the proportions of substitutions, deletions and insertions described in^12^ (high mutagenesis), with a multiplier between 0.1 and 2.2. The output constraints used were a maximum homopolymer length of 3 and, for the codes that support it, a GC content between 40 and 60% in 10 nt intervals. The last ratio, in which the code had a 100% success rate (out of 100 repetitions) and up until the first time, the code had a zero percent success rate, is shown for each code. Points represent the results of the simulations, while dashed lines are interpolated values. Source data are provided as a Source Data file.

Since all error-correcting codes evaluated in this work can correct substitution errors, we first evaluated the implementations regarding their ability to correct such errors. We encoded a 4.8 KB text file containing the German version of the fairy tale Dornröschen (sleeping beauty). We used each code and inserted substitution errors at random positions in the encoded files. We gradually increased the number of errors in steps of 500 substitutions and repeated for each point the process 100 times, each time with randomly chosen error positions. Löchel and Heider presented a similar approach^9^. The results of the simulations are shown in Fig. 1a, with the number of successful decoding attempts plotted against the number of substitutions per encoded base (the base error ratio, BER). The code parameters used for the simulations are described in the supplement. We constrained the encoder output not to include homopolymers longer than three bp to be consistent for all codes to the fixed homopolymer length of the Grass code. We also used the common constraints of a GC content between 40 and 60%^6,7,21,22^ in 10 bp intervals for all codes that support it. As an additional constraint for DNA-Aeon, it had to have the lowest total number of encoded bases of all codes evaluated. DNA Fountain successfully decoded the data 100% of the time up to a BER of 0.006, with a rapid decline in successful decoding attempts afterwards, reaching zero percent at a BER of 0.016. For the Grass code, we observed a successful decoding rate of 100% up to a BER of 0.012, with a 94% success rate at a BER of 0.018, followed by a rapid decline to zero percent at a BER of 0.024. For Hedges, we observed a 100% success rate, up until a BER of 0.031, with a 98% success rate at 0.039. At a BER of 0.046, Hedges still had a success rate of 77%, with a steeper decline afterwards, reaching 42% decoding success at a BER of 0.054 and zero percent at a BER of 0.077. DNA-Aeon was able to successfully decode the input data 100 % of the time up to a BER of 0.07, with a slow decline to 95% decoding success at a BER of 0.077, and a sharp drop-off to 0% decoding success at a BER of 0.85. Tables of the results are available in the supplement.

Since the processes involved in DNA data storage not only lead to substitution errors but also insertions and deletions (indels), we used the MESA error simulator^8^ to simulate realistic storage conditions, including indels. We used the pre-configured error rates of MESA for array-based oligo synthesis^23^ and Illumina paired-end sequencing^24^, together with depurination at pH 8 and 253.15 K for 120 months to simulate in vitro storage. Besides the Grass code, which does not account for indels and returns an error if the sequence length is not a multiple of 3, each code was able to decode the input data successfully. To better compare DNA Fountain, Hedges, and DNA-Aeon in the presence of indels, we used the error rates that were observed by the Hedges authors when using a high mutagenesis kit^12^ as a baseline, with a substitution rate of 0.0238, a deletion rate of 0.0082, and an insertion rate of 0.0039. Beginning with a multiplier of 0.1, we tested the three codes that can account for indels with the error rates, the multiplier and the output constraints described above. We gradually increased the multiplier in steps of 0.1 and repeated the simulation 100 times for each multiplier. The results are shown in Fig. 1b. Since DNA Fountain did not have a 100% success rate with a multiplier of 0.1, we did an additional evaluation with a multiplier of 0.05 for DNA Fountain to reach a 100% successful decoding rate. DNA Fountain was able to correct 100% of the errors at a BER of 0.002, and 41% at a BER of 0.01, with a rapid decline to 0% at a BER of 0.014. Hedges decoded the input data correctly 100% of the time up to a BER of 0.036, a 95 % success rate at 0.4, followed by a decline in successful decoding attempts, with a success rate of 56% at a BER of 0.047 and 16% at a BER of 0.054. The success rate reached 0% at a BER of 0.061. DNA-Aeon was able to correctly decode the input data 100% of the time up to a BER of 0.065, with a success rate of 99% at a BER of 0.068 and 77% at a BER of 0.072. The success rate sharply declined to 4 % at a BER of 0.075, followed by a 0% success rate at a BER of 0.079. However, given that the error-correcting capabilities of Hedges change depending on the leniency of the output constraints, more lenient output constraints (longer homopolymer chains or a more varying GC content in a broader window) would increase the error-correcting capabilities of Hedges. In contrast, stricter output constraints would reduce it. Furthermore, DNA-Aeon constrains the GC content in intervals, while Hedges constrains the GC content in sliding windows.

### Rate comparison

**Fig. 2:**
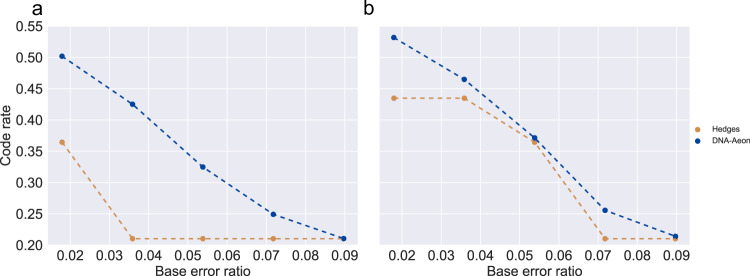
Code rate comparison: required code rate (information nucleotides divided by total nucleotides encoded) for successful decoding 100 out of 100 tries for a given BER. **a** using the output constraints of a GC content of 40–60% in 10 bp long intervals and a maximum homopolymer length of 3, **b** with output constraints of a GC content of $33.\overline{3}\%$ to $66.\overline{6}\%$ in 12 bp long intervals and a maximum homopolymer length of 4. Points represent the results of the simulations, while dashed lines are interpolated values. Source data are provided as a Source Data file.

We analyzed the two best-performing codes of the previous comparisons (DNA-Aeon and Hedges) to evaluate the relationship between redundancy and error correction capabilities. For this purpose, we used the 4.8 KB text file and the high mutagenesis frequencies described in the previous section and gradually increased the multiplier in steps of 0.5, up to 2.5 times the observed values. This translates to an error rate of up to 9 %, the equivalent of the expected amount of degradation after 150 years of storage in nature (i.e., in buried bones at 13 ^∘^C) for 100 bp long sequences^25,26^ and the highest recommended error rate for DNA-Aeon. Under optimal conditions (encoded DNA embedded in silica particles and stored at −18 ^∘^C), the error-correction performance of DNA-Aeon would allow the storage of data in DNA for millions of years^10^. We adjusted the parameters of the evaluated codes to achieve error-free decoding in 100 out of 100 times with the minimal amount of encoded bases possible for each step. The analysis was carried out for a GC content of 40–60% in intervals of 10 bp and with no homopolymers longer than 3 nucleotides (Fig. 2a), and also for Hedges default constraint parameters, comprised of a GC content between $33.\overline{3}\%$ to $66.\overline{6}\%$ in 12 bp windows and a maximum homopolymer length of 4 (Fig. 2b). For all error rates evaluated here, DNA-Aeon was able to retrieve the encoded data error-free using less bases than Hedges. Especially for low error rates, typically observed in DNA data storage^4^, DNA-Aeon requires considerably less redundancy than Hedges. For the analysis carried out with a GC content of 40–60% in intervals of 10 bp and with no homopolymers longer than 3, DNA-Aeon requires 27% fewer bases than Hedges if a 0.5 multiplier is applied to the error rates observed by Press et al.^12^ using a high mutagenesis kit (which translates to an error rate of 1.8%). For the moderate error rates evaluated, using a multiplier of 1.0 (an error rate of 3.6%) and 1.5 (an error rate of 5.4%), DNA-Aeon required 51% and 35%, respectively, fewer bases than Hedges. For the high error rates evaluated, using a multiplier of 2.0 (an error rate of 7.2%) and 2.5 (an error rate of 9%), DNA-Aeon required 16%, and <1%, respectively, fewer bases than Hedges. For the analysis carried out with Hedges default constraint parameters, DNA-Aeon requires 18 % less bases using a 0.5 multiplier (error rate of 1.8%), 6 % less bases using a 1.0 multiplier (error rate of 3.6%), 2% less bases using a 1.5 multiplier (error rate of 5.4%), 18% less bases using a 2.0 multiplier (error rate of 7.2%) and 1.8% less bases with a 2.5 multiplier (error rate of 9%). The Hedges parameters used for the evaluations are described in the supplement, and the DNA-Aeon configuration files used for the evaluations are available in the DNA-Aeon GitHub repository.

### Cost analysis under realistic conditions

One major disadvantage for the large-scale adoption of DNA as a data storage device is the high cost of DNA synthesis. Thus, apart from providing good error correction performance, the cost efficiency of codes should be investigated.

**Fig. 3:**
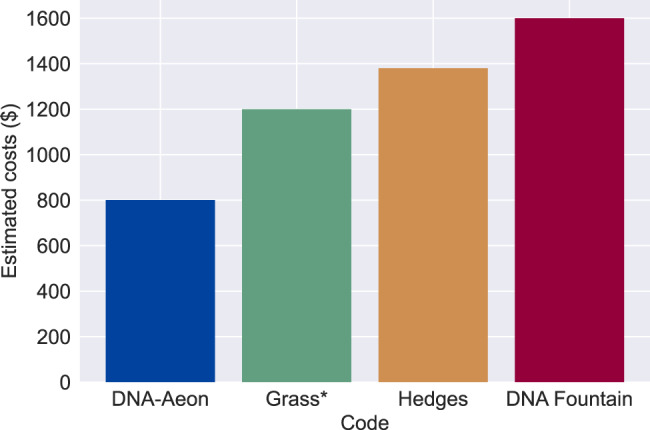
Cost evaluation: estimated synthesis costs of encoding a 4.8 KB file with enough redundancy to successfully decode it after synthesizing, in vitro storage for ten years, and sequencing. The Grass code cannot decode the data in the presence of insertions or deletions. The simulations were carried out for each code 10 times. Source data are provided as a Source Data file.

To evaluate the cost efficiency, we used error rates as described in the literature for array-based oligo synthesis^23^ and Illumina paired-end sequencing^24^, together with depurination at pH 8 and 253.15 K for 120 months to simulate in vitro storage. The error rates are available as supplemental file. In our simulations, we used the DNA error simulator MESA^8^. We encoded a 4.8 KB text file containing the fairy tale Dornröschen, using the four codes described earlier. When possible, we adjusted the parameters of each code to allow the decoding of the input data in the presence of errors simulated by MESA, with as little redundancy as possible. The chosen parameters are available in the supplement. We used the oligo pool pricing table of Twist Bioscience to estimate the costs of synthesizing the encoded data. The results are shown in Fig. 3. DNA-Aeon can decode the data using 294 strands of 114 bases each, for a total of 33,516 bases. Hedges needs 255 strands of 210 bases each, for a total of 53,550 bases. The Grass code requires 713 strands of 118 bases each, leading to a total of 84,134 bases, while DNA Fountain requires 1500 strands of 76 bases each, totaling 114,000 bases.

### mCGR evaluation

**Fig. 4:**
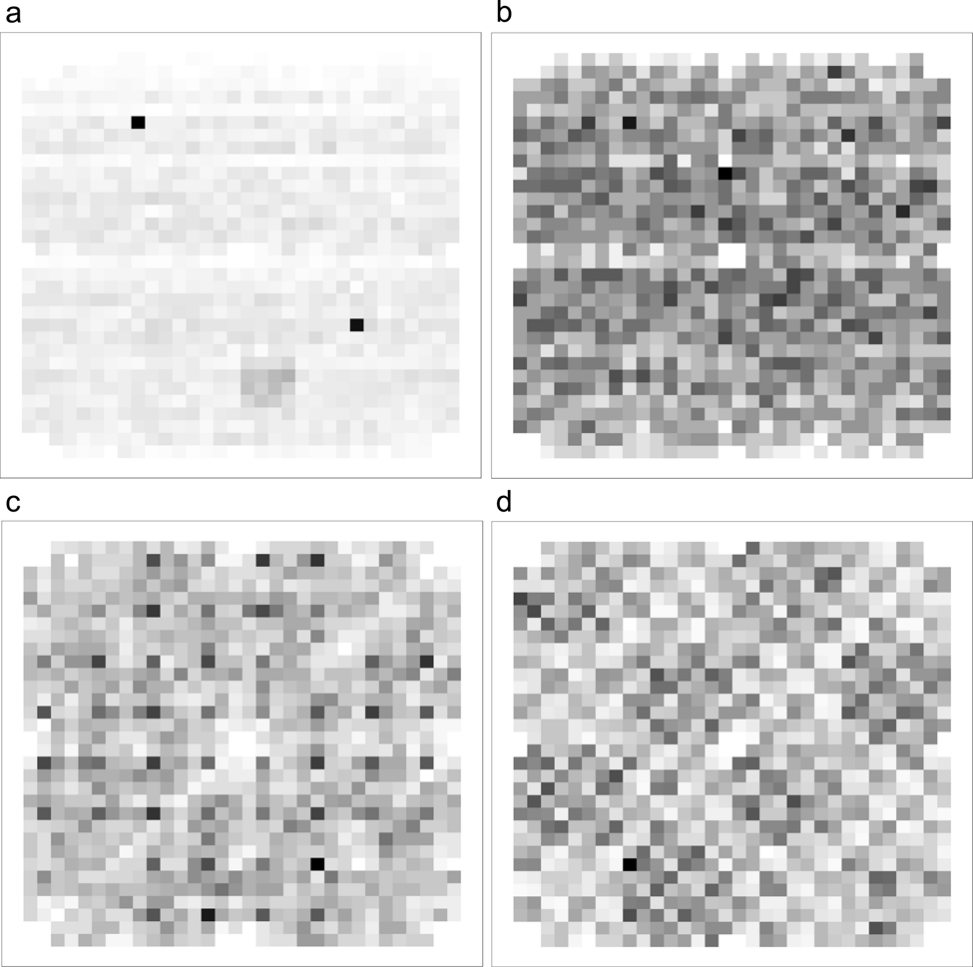
Sequence fragment distributions. mCGR representing the frequency of all possible sequences of length 5 for Hedges (**a**), DNA-Aeon (**b**), Grass code (**c**) and DNA Fountain (**d**). Source data are provided as a Source Data file.

To evaluate the distribution of sequence fragments, we evaluated all four encodings with mCGR (matrix chaos game representation)^2^ and the R package kaos^27^, as described in the supplement. The mCGR is based on the Chaos Game Representation, which arranges DNA sequences in fractal patterns and has, therefore, multiple applications in bioinformatics and computational biology^28^. To this end, we carried out an mCGR analysis of a 4.8 KB text file containing the fairy tale Dornröschen, encoded using the codes described above. We split the encoded sequences into fragments of length 10. The mCGR for *k* = 10 represents the frequency of all possible sequences in the length of 10. We decreased *k*, which leads to a clustering of sequences with the same postfix. The results for *k* = 5 are shown in Fig. 4, and the results for different *k* are available in the supplement. While the nucleotide composition of the encoded data is equally distributed for DNA-Aeon, a chessboard-like pattern in the DNA Fountain encoded data can be observed. In the mCGR of the Grass code, a cross-like pattern emerges, resulting from a high presence of dimers of the same nucleotide. In the Hedges encoded data, an overrepresentation of the sequence GTA, TGC, and GTC in the form of clusters can be observed. The results indicate that DNA-Aeon can better exploit the possible code space. In addition, since DNA-Aeon can incorporate a user-defined codebook based on mCGR, it would be possible to also encode meta-information in the codebooks. Moreover, the mCGR analysis can be used to identify the underlying code in case this information is lost, e.g., when information is stored for several years.

### In vitro results

**Fig. 5:**
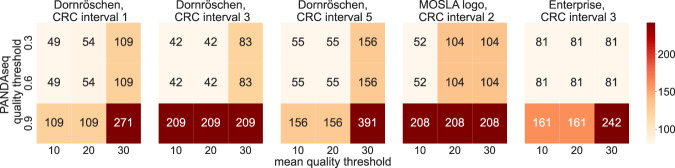
Required amount of sequencing reads: the minimal amount of raw sequencing reads, as a multiple of the amount of encoded data, that were required for successful decoding under various raw read processing parameters and CRC intervals. The CRC intervals represent the number of information bytes between two 8-CRCs in the encoded data, the mean quality threshold is the minimal average quality score for a raw read to not be discarded during processing. In contrast, the PANDAseq quality threshold represents the quality an assembled sequence (from the corresponding forward- and reverse-read) has to achieve to not be discarded. The evaluation was carried out a single time for each parameter combination, file, and read percentage. Source data are provided as a Source Data file.

To validate the ability of DNA-Aeon to decode data stored in DNA, we encoded three different files of size 4.8 KB (a text file containing the German version of the fairy tale sleeping beauty, Dornröschen), 29.9 KB (a PNG of the logo of the MOSLA research cluster), and 47.1 KB (a JPEG of the Enterprise NCC 1701-D) with different parameters. We synthesized the encoded data, followed by PCR amplification and sequencing to digitize the DNA. Information regarding the encoded files, parameters used, and biological processing can be found in the supplement. The raw sequencing data was then processed using parts of the read processing pipeline Natrix^29^. Since it is common in read processing pipelines to have various quality control steps in which reads that do not reach a quality threshold are discarded, we tested different quality thresholds during initial quality control and after the assembly of paired-end reads. The initial decoding was successful for all inputs using 100% of the FASTQ data. We gradually reduced the percentage of the raw reads used for processing and decoding until the decoding failed. The different properties of the last successful decoding for each parameter combination are provided in the supplement and shown in Fig. 5. Using a lower quality threshold both before and after assembly led to less raw sequencing data needed for decoding, while the amount of processed sequencing data needed for successful decoding was between 1.1 and 1.96 times the encoded data. We furthermore tested the influence of adding a 97% similarity clustering as the last step of the processing before encoding. In the clustering approach, sequences are ordered in a list by abundance, with the most common one first, serving as the representative sequence of the first cluster. All sequences that are at least 97% similar to the first sequence are added to this cluster and removed from the list of sequences. This process repeats until no sequences are left in the list, with only the representative sequences being further evaluated. The results are shown in the supplement. The clustering doubled the number of raw sequences required for the MOSLA logo at 0.3 pq, 20 mq, and for 0.6 pq, 20 mq (with mq = the mean minimal quality of reads to not be discarded, and pq = minimal quality of the read assembly to not be discarded). For every other parameter combination, no changes in the amount of required raw sequences were observed. The similarity clustering led to a general decrease in required processed sequences to decode the data, as only 0.741–0.966 times the encoded data were required for successful decoding. The decreased amount of processed data needed for decoding using similarity clustering, while the number of raw sequences required remained the same compared to no clustering for most cases, implies that the clustering led to a decrease in redundant sequences without improving the error correction performance. Given the variation of mapped reads per sequence in the FASTQ files (supplemental Table 8), with some sequences only constituting 0.001% of the raw FASTQ files, DNA-Aeon was able to decode the data with only 0.9–10% of the raw FASTQ data.

## Discussion

DNA-Aeon is a flexible code for DNA data storage that can be used to encode data in DNA that adheres to a variety of constraints. The codebook approach for constraint adherence supports the usage of DNA-Aeon for different synthesis, storage, and sequencing method stacks and easily interfaces with tools for the generation of codebooks. The provided codebook tool^2^ can adhere to the common constraints of variable GC content, homopolymers of variable lengths, and undesired motifs. Compared to other codes, DNA-Aeon encoded data shows no discernible nucleotide distribution patterns, increasing the resilience of the encoded data against errors. Furthermore, DNA-Aeon can correct substitution, deletion, insertion errors, and the complete loss of DNA fragments to a high degree. The user can set several parameters of the decoder using a configuration file. Each parameter is explained in detail, allowing further customization and improvements in error correction capabilities according to the used synthesis, storage, and sequencing methods and the expected error probabilities. In the event of unexpected high error occurrence not accounted for during encoding, the stack size and the number of stack removals executed can be adjusted. This flexibility facilitates further improvements of error correction capabilities at the cost of increased memory consumption or runtime. Furthermore, the flexibility of both fragment size and amount of fragments generated can be leveraged to encode data in DNA. This data can be successfully decoded in the presence of error rates described in the literature for synthesis, storage, and sequencing at <60% of the costs of other codes, thus paving the way for an economic use of DNA storage systems. Finally, even with sequencing data with a highly skewed coverage distribution, DNA-Aeon can decode data without extensive read processing.

## Methods

### Code design overview

**Fig. 6:**
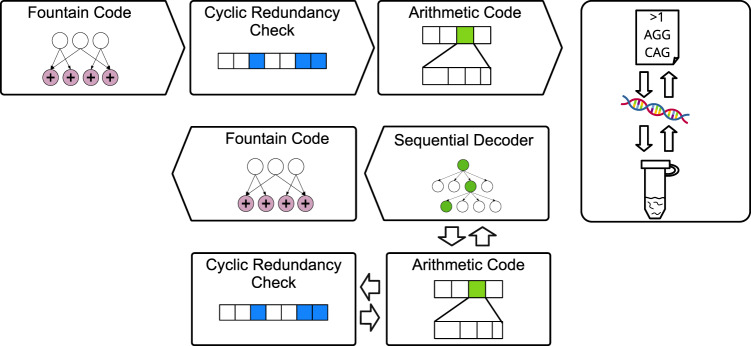
Overview of the DNA storage workflow using DNA-Aeon: Input data is encoded and packetized using the NOREC4DNA Raptor fountain code, followed by periodic insertion of an 8-bit CRC checksum, including a final CRC to protect the end of the packet. The packets are then encoded in parallel using the arithmetic code, using a constraint-free codebook. The channel (right side of the figure) represents the DNA synthesis, storage, and sequencing of the encoded data. The channel output packets are decoded in parallel by the inner, sequential decoder. The sequential decoder stores the states of the arithmetic code as nodes. In periodic intervals, a CRC check of the data that was decoded since the last CRC checksum is performed. The fountain code then uses the packets with the highest final Fano metric to recover the original input data.

Our code consists of an outer fountain code and an inner code that resembles an arithmetic code, with a switched en- and decoder (Fig. 6), i.e., our encoder uses principles of arithmetic decoding. The outer code uses the Raptor fountain code implementation of NOREC4DNA^7^ in the binary mode, which can generate all possible packets in a given seed range. Users can define either the number of packets generated from a file or the size of the individual packets generated. Furthermore, the addition of an optional header chunk is supported, containing meta-information such as filename, permissions, and padding of the last packet.

The inner encoder takes as its input the packets generated by the outer code, and a FASTA file and concatenation scheme of codewords. One option for the generation of the additional input is the mCGR approach of ConstrainedKaos^2^ tool, which generates codewords with user-defined constraints, namely GC content, homopolymers, and undesired motifs. A model with transition probabilities is generated using the codebook. In essence, the inner encoder is an arithmetic decoder, treating the input as a compressed representation of a constraint-adhering DNA sequence. Using source decoding to encode data into a run-length limited representation was previously described by Dubé et al.^30^. Each fountain encoded packet is logically split into subpackets of the same, user-defined size, and a Cyclic Redundancy Check (CRC) is assigned to each subpacket. These CRCs serve as verification and synchronization markers for the decoder, increasing the substitution correction performance and allowing the correction of synchronization errors (insertions and deletions). The approach of using marker symbols for error detection in joint source and channel coding was previously described by Elmasry^31^.

### Arithmetic coding principles

**Fig. 7:**
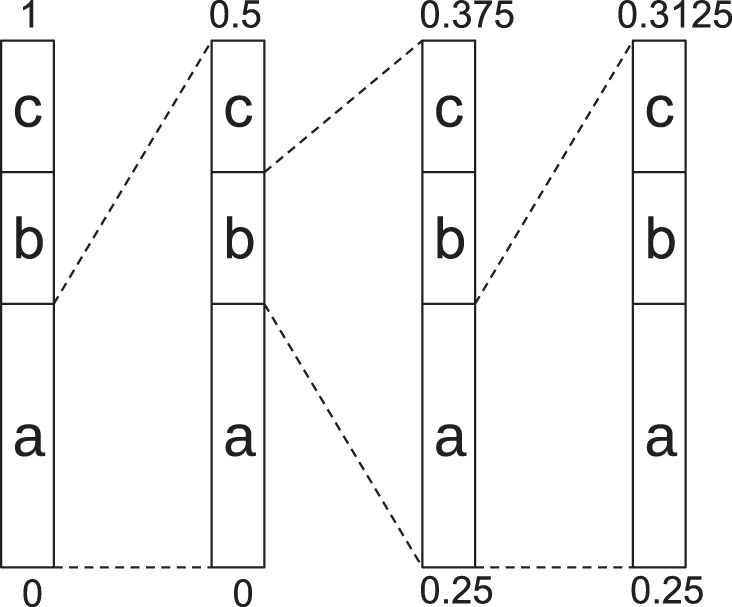
Arithmetic coding example: encoding schematic of the string abac with probabilities *a*: 0.5, *b*: 0.25, *c*: 0.25. After encoding the final symbol c, the final subinterval is [0.296875, 0.3125).

Arithmetic coding is a lossless entropy encoding technique used as a basis for many common video standards^32^. It compresses data by iteratively partitioning the interval [0, 1) into smaller subintervals. The partitioning of the current interval depends on symbol probabilities given by a model. In the simple case, the current interval is split into subintervals whose length is proportional to the probability of a symbol occurring in the data. For a string abac, the probabilities would be *a*: 0.5, *b*: 0.25, *c*: 0.25, and in the first iteration, the interval [0, 1) would be split into the subintervals [0, 0.5), representing a as the first symbol of the string, [0.5, 0.75), representing b, and [0.75, 1) representing c as the first symbol of the string. As a is the first symbol in the example, the current subinterval is [0, 0.5) after encoding the first symbol. It will be divided into the subintervals [0, 0.25), [0.25, 0.375), and [0.375, 0.5) in the next iteration. Figure 7 shows the complete encoding process of this example (the final subinterval being [0.296875, 0.3125)). Every real number in the final interval can be used as the compressed representation of the input, with the number having the smallest bit string representation being commonly chosen. The decoding of arithmetically encoded data follows the same steps as the encoding, with the encoded data as input: starting in the interval [0, 1), the decoder checks in which subinterval the encoded data falls. In the example above, every real number of the final subinterval [0.296875, 0.3125) of the encoding first falls into the subinterval [0, 0.5), representing a in the first iteration, in the second iteration, [0.25, 0.375), representing b, in the third iteration [0.25, 0.3125), representing a, and in the final iteration [0.296875, 0.3125), representing c.

Arguably the most crucial part of an arithmetic code is the model: a model that accurately matches the actual symbol probabilities has a higher compression rate than a model that does not. One possibility to increase the accuracy of the model is to utilize an adaptive model, in which the symbol probabilities in each iteration change according to the data that was previously encoded.

### Arithmetic modulation

In our approach, we treat the input data as a binary, compressed representation of a DNA string. As explained above, the encoding follows the principle of arithmetic decoding, with a model generated using a set of allowed DNA codewords. During each iteration of the encoding process, the model divides the four possible subintervals, each representing one of the four bases A, T, G, and C, according to occurrence probabilities yielded by the model. This approach enables the transcoding of binary data into constraint-adhering DNA sequences. Utilizing the principles of arithmetic decoding to encode binary data into constraint-adhering DNA sequences also allows the detection of errors during decoding. Decoding fails if an error occurs that violates the codebook’s constraints (i.e., a base with no subinterval assigned to it in the current decoder state). While this approach can detect some errors, it is not able by itself to pinpoint the exact location the error occurs. For example, if the decoding failed because of a homopolymer that exceeded the maximum length allowed by exactly one base, each of the homopolymer members could be the erroneous base. To incorporate a consistent ability to detect and correct substitutions and indels during the encoding process, an 8-bit long CRC is periodically inserted into the encoding stream. This CRC is calculated from the input bytes that were processed by the arithmetic encoder since the last incorporation of a CRC. The periodic insertion of CRCs allows the detection and subsequent correction of synchronization errors (insertions and deletions) and substitutions. Since the iterative narrowing of the code interval leads to severe error propagation, a single wrong, missing, or inserted base leads to vastly different decoded sequences. It will therefore lead to the failure of multiple CRC checks. Since the model returns base frequencies for each base and position, a potential erroneous base can be replaced by a base with a high codebook frequency (i.e., a high probability that the base was inserted at this position by the encoder) at the position if an error is detected.

The user can freely choose the interval between two CRCs (as the step-size parameter *s*), adjusting the code-rate depending on the anticipated noise of the storage channel.

### Model generation

**Fig. 8:**
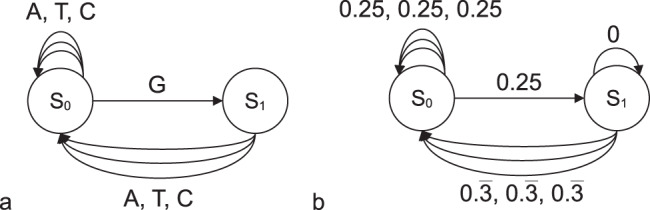
State transition example for sequence constraints. With two states, *S*_*0*_ and *S*_*1*_, and four possible inputs (A, T, C, and G) (**a**) that map to model probabilities (**b**): finite state transition diagrams for a model in which two consecutive G’s are forbidden (**a**) and probabilities as returned by the model (**b**).

The basis for our model is a codebook file containing DNA strings of uniform length in the common FASTA format. In addition, a concatenation scheme is required. This concatenation scheme is a JSON file containing key-value pairs of codeword prefixes and suffixes that are not allowed to match in the encoded data. The GitHub repository contains codebooks and concatenation schemes for common constraint combinations. Custom codebooks can be generated using the ConstrainedKaos^2^ or other codebook generation tools. A model in the form of a finite state transition diagram (FSTD) with transition probabilities is generated by DNA-Aeon, using the frequencies of the bases at each position of the codewords. A simple finite state transition diagram, in which two consecutive G’s are not allowed, is shown in Fig. 8. Each time the encoder reaches a multiple of the codeword length, the FSTD returns to the first state, with some transitions disabled according to the concatenation scheme. This prevents that the encoded data contains undesired motifs or homopolymers that form between two codewords without the need to discard all codewords that contain pre-/suffixes that could form such sequences. With increasingly stringent constraints, the number of available codewords shrinks, leading to an increase of redundancy introduced by the encoder, as shown in (1), where *C* is the number of valid codewords and *n* is the length of each codeword.

1 $${{{{{{{\rm{redundancy}}}}}}}}\left(\frac{{{{{{{{\rm{bit}}}}}}}}}{{{{{{{{\rm{base}}}}}}}}}\right)=2-\frac{{\log }_{2}(|C|)}{n}$$

To estimate the encoded sequence *b* from a channel output *y*, maximum a priori (MAP) estimation can be used, utilizing the redundancy introduced for constraint adherence.

### Estimation metric

The redundancy introduced by the encoder can be exploited by the utilization of MAP estimation to find the most likely encoded sequence *b* out of a set of possible sequences Ω and from a known channel output *y*:

2 $${\hat{b}}^{{{\mbox{MAP}}}}	=\arg \mathop{\max }\limits_{b\in \Omega }P(b|y)\\ 	=\arg \mathop{\max }\limits_{b\in \Omega }\frac{P(y|b)P(b)}{P(y)}$$

The Fano metric^33^ can be derived from the MAP sequence estimator, as shown by Moon^34^. This metric is a cumulative, symbol-by-symbol path metric for approximating the maximum likelihood path through a decoding tree without evaluating all possible paths. For a binary symmetric channel with transition probability *p*_*t*_ and code rate *R*, the Fano metric for the channel output symbol *y*_*i*_ is

3 $$\mu ({y}_{i},{b}_{i})=\left\{\begin{array}{ll}{{{\mbox{log}}}}_{2}(2(1-{p}_{t}))-R &\,{{\mbox{if}}}\,\,{y}_{i}={b}_{i}\\ {{{\mbox{log}}}}_{2}(2{p}_{t})-R\hfill &\,{{\mbox{if}}}\;{y}_{i}\,\ne\, {b}_{i}\end{array}\right.$$

The Fano metric consists of two parts: a correctness term that adds log_2_(2(1 − *p*_*t*_)) to the path metric if the candidate base agrees with the channel output base at the current position, and a bias term *R*, which is subtracted from the total metric for each base once. The bias term functions as a path-length equalizer, allowing the comparison of paths of different lengths. If only paths of the same length are compared, the bias term is the same for all paths. In contrast, if paths of different lengths are compared, longer paths have a larger bias, offsetting the potentially higher path metric as more positions in the channel output could be evaluated in the longer paths. The metric makes use of binary symmetric channel model properties^34^, which differs from the DNA data storage channel^35^ and serves, therefore, as an approximate metric for DNA data storage coding. We have added an additional term to the Fano metric, adding the prior probability of the base (i.e., the probability that the base was added at this position by the encoder) to each case. As a result, a path with a base that did not agree with the channel output, but has a high probability of being added by the encoder at this position has a higher metric than a base that did not agree with the channel output with a low encoding probability. If a decoding failure of the current candidate sequence takes place, the path of the base with the higher encoding probability can be evaluated as a new candidate sequence. This symbol-by-symbol metric, utilizing encoding probabilities, can be used for tree-based decoding.

### Decoding

**Fig. 9:**
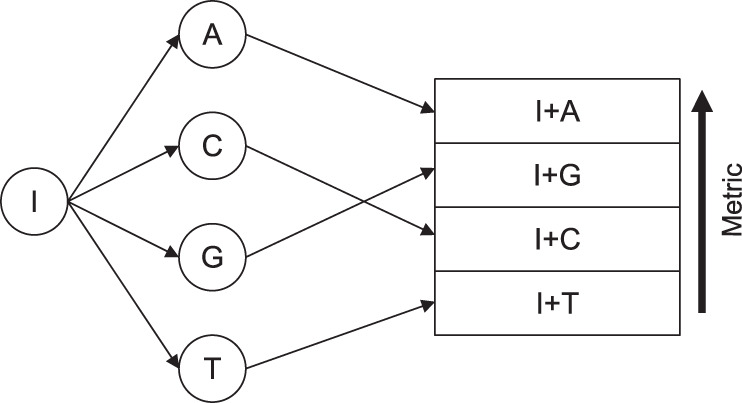
Principle of one iteration of the stack algorithm for DNA: an input node *I* is extended for each of the possible bases (A, C, G, and T) at the current position. The new decoding branches (*I*+A, *I*+G, *I*+C, and *I*+T) are then inserted into the decoding stack, and the stack is sorted according to the branch metrics. The node(s) with the highest metric in the stack will be extended in the next iteration.

Our decoding process utilizes a variation of the stack algorithm^36,37^. The stack algorithm is a sequential decoding algorithm in the form of a decision tree that keeps an ordered stack with size *M* of decoding paths stored. Compared to decoding algorithms like the Viterbi algorithm^38^, the stack algorithm only evaluates the most likely candidate sequence at each decoder iteration. The algorithm’s sequential nature allows using arithmetic demodulation states as decoding tree nodes. For a channel output sequence *y*_1:*v*_, the path corresponding to the most likely encoded sequence ${\hat{b}}_{1:g}$ will be on top of the stack. The adjusted Fano metric serves as an evaluation metric for sorting the stack. After each decoding iteration, the decoding path with the best metric is removed from the top of the stack and extended to multiple branches. The decoding path is extended once for each possible next symbol, with branches that lead to symbols that are not possible, according to the model, being removed. Each of the new nodes is then inserted into the decoding stack, after which the stack is sorted according to the branch metrics (Fig. 9). A node of the decoding tree represents a decoding state (*X*_*n*_, *K*_*n*_), with *X*_*n*_ as the internal state of the arithmetic decoder and *K*_*n*_ as the number of symbols that can be decoded in this state^39^. Each time *K*_*n*_ reaches a multiple of the step-size parameter *s*, a CRC validation of the last *s* decoded bytes is executed. If the validation fails, the node with the failed CRC is removed, and the corresponding path will not be further evaluated. This removal mechanism reduces path evaluations, increasing the probability that the correct path will remain in the stack. The arithmetic decoding process leads to delays between an erroneous symbol *y*_*n*_ entering the decoding register and the output of a wrongly decoded symbol^40^. To account for this delay, we further added a penalty parameter to all nodes of a decoding subtree spanning from the same CRC node if multiple paths of this subtree failed the subsequent CRC check. This approach decreases the number of evaluations in a local optimum, i.e., it offsets the higher metric that longer sequences get in the case of multiple CRC failures. After the top decoding branch reaches a predefined length, a final CRC check is carried out to validate the integrity of the complete decoded sequence, followed by the termination of the decoding process if the final CRC validation was successful. If the stack size reaches *M*, the decoding branches with the worst metric are deleted from the stack. This stack removal mechanism reduces the memory requirements of the algorithm, and the user can freely choose *M*. To account for insertions and deletions, the decoding branch with the best metric is extended to up to nine branches, four branches that estimate the next base as *A*, *T*, *C* or *G*, four branches that assume a deletion has taken place, and one branch that assumes an insertion has taken place if ${\hat{b}}_{v}={y}_{v+1}$. Furthermore, we allow the user to set the number of nodes that are removed from the top of the stack and subsequently extended, as a form of a generalized stack algorithm^41^.

### NOREC4DNA Raptor fountain code concatenation

While the code described here is functional on its own, we concatenated it with the NOREC4DNA implementation of a Raptor fountain code^7^ as an outer code. Since fountain codes can generate numerous (depending on the implementation, up to infinite) packets from the input data, users can freely adjust the rate of the outer code depending on the anticipated error probabilities, the intended purpose, and the budget. The fountain encoded data can be decoded in any order; thus, it is not required to add indices to the data by the inner encoder. Instead of indices, a seed is used. With this seed, the encoder samples a distribution function to retrieve the number of chunks (*n*) that will be XORed into the packet and then uses the same seed to choose these *n* chunks. The decoder can reconstruct which chunks were used for each encoded packet by applying the seed to the same distribution function. With this information, the decoder can reduce multiple packets to the original chunks using either belief propagation or Gaussian elimination with partial pivoting. The decoding process only needs (1 + *ϵ*) ⋅ *n* correct symbols to decode the input data. We utilized this feature of fountain codes by adding the final metric of each packet decoded by the inner decoder to the data before passing it to the outer Raptor-based fountain code, which then uses the final metric to choose the packets used for the decoding procedure. In contrast to the previously widely used LT-based fountain code for DNA data storage^11^, we chose a Raptor-based encoding to benefit from the greatly reduced required overhead, stability, and significantly decreased susceptibility to the coupon collectors problem^7^. Using a fountain code as an outer encoder enables the reconstruction of the encoded data if a sequence fragment is too damaged for the inner code to repair or in the case of a loss of complete fragments. NOREC4DNA allows the analysis of an optional checksum of each received packet during the decoding. If the checksum indicates that the packet is corrupt, it will be discarded in this step. In addition, the outer encoding adds a header chunk containing metadata such as the filename and an additional file-wide checksum. After a successful reconstruction, this checksum is used to verify the integrity of the decoded data. In the case of a mismatch, the packet overhead can be used to reconstruct the file. For this fallback, the decoder reorders the packets in the (over-complete) Gaussian elimination equation. This approach can reconstruct the file without errors if a solution exists in which the corrupt packet(s) are not required. This approach works even if there is no packet-level checksum or if a packet-level checksum collision exists. The packet approach of fountain codes, in which each sequence strand is treated as an individual packet, and the ability to adjust the redundancy by increasing the number of packets, gives users enhanced flexibility in using DNA-Aeon for their specific needs. For example, with this approach, generating a large number of packets is possible, followed by screening the encoded packets according to a user’s needs. A user that requires the encoded packets to have a very low probability of secondary structure formation at a specific temperature could use a tool to screen the encoded packets (e.g., MESA^8^) and only use packets that satisfy these requirements. Another example of the increased flexibility provided by using a Fountain code as the outer code is the ease of generating new packets. If specific packets are not desirable (e.g., if the integration into a vector or the host genome for in vivo storage fails or unforeseen difficulties during the synthesis arise), fountain codes allow users to additionally generate new packets without requiring to replace all prior encoded packets. Finally, the processes of synthesis, PCR, storage, and sequencing can lead to the loss of complete packets^11^, and the ability of Raptor fountain codes to reconstruct the original data as long as any (1 + *ϵ*) ⋅ *n* of the encoded packets are present makes it well suited as the outer code of DNA-Aeon. Since other error-correcting codes (e.g., Reed-Solomon codes) can be used as outer codes, it would be interesting to evaluate their properties compared to fountain codes in future studies.

### Reporting summary

Further information on research design is available in the Nature Portfolio Reporting Summary linked to this article.

## Supplementary information

Supplementary Information

Reporting Summary

## Data Availability

Sequence data that support the findings of this study have been deposited in the sequence read archive under accession codes SRR19954693, SRR19954695, SRR19954696, SRR19954697 and SRR19954694, BioProject accession PRJNA855029. The error-correction and rate analysis data generated in this study are provided in the Supplementary Information and Source Data files. The raw data of the sequence processing parameter evaluations are provided in the Source Data files. The MESA configuration file used for the cost analysis under realistic conditions is provided in the Source Data files. The final parameters used for the rate analysis are available in the Source Data files. The encoded data, split into 10mers, as used for the mCGR evaluation, are available in the Source Data files. No data restrictions apply. Source data are provided with this paper.

## Source data

Source data

## Code availability

The source code of DNA-Aeon is available at https://github.com/MW55/DNA-Aeon. The version used in this work is version 1.0^42^.
