# Supplementary material for: DNA-Aeon provides flexible arithmetic coding for constraint adherence and error correction in DNA storage
Source: Nat Commun. 2023 Feb 6;14:628. doi: 10.1038/s41467-023-36297-3 (PMC9902613; doi:10.1038/s41467-023-36297-3)
Supplement: Supplementary file 1 — Supplementary Information [file 41467_2023_36297_MOESM1_ESM.pdf]

Supplementary Material for: DNA-Aeon Provides Flexible Arithmetic Coding for Constraint Adherence and Error Correction in DNA Storage

Marius Welzel, Peter Michael Schwarz, Hannah F. Löchel, Tolganay Kabdullayeva,
Sandra Clemens, Anke Becker, Bernd Freisleben, and Dominik Heider

January 2023

1 Installation and configuration of the codes evaluated in this work

1.1 Code by Grass et. al.

Installation

The code described by Grass et. al. [1] was downloaded from https://github.com/reinhardh/dna_data_storage, commit e701abc (the most recent as of April 12, 2022). The installation took place as described in the accompanying README file. To compile the program, we removed one redundant class identifier in ReedSolomon.hpp, line 160, as it led to a compile error under g++ 11.2.0.

Configuration

Since the Grass code does not offer customization in the form of different parameters, its usage is straightforward. We encoded data using the command:

```
$ <path>/texttodna --encode --input=<input file> --output=<output file>
```

The only parameter that can be adjusted for decoding is the number of blocks that were generated by the encoder. In our evaluations, using a text file with a size of 4.8 KB, the number of blocks generated was 1. The decoding command is as follows:

```
$ <path>/texttodna --decode --numblocks=1 --input=<input file> --output=<output file>
```

1.2 Hedges

Installation

Hedges [2] was downloaded from https://github.com/whpress/hedges, commit c85ccb8 (the most recent as of April 12, 2022). The instructions of the README file could not be used for installation, since the repository neither contained a requirements.txt, nor a setup.py file; both are referenced in the installation instructions. We instead created a python 2.7 virtualenv environment with numpy 1.13.3 to use the Python wrapper and used the pre-compiled binaries of the C++ code.

Configuration

In the evaluations using a fixed set of parameters, we used the code rate of 0.5, since this was the highest code rate that allowed the encoding of the 4.8 KB test file in a single block. Lower code rates led to decreased error correction performance (to the point of decoding failures in the absence of errors) or program crashes. The Python wrapper of Hedges requires the presence of primers. Since no other code evaluated requires primers, we reduced the size of the primers to a single base each (A as a forward primer and C as a reverse primer)

Multiplier	coderatecode	totstrandlen
0.5	2 (0.6)	176
1.0	2 (0.6)	176
1.5	3 (0.5)	210
2.0	4 (0.33)	182
2.5	4 (0.33)	182

(a) GC $33.\overline{3}$ - $66.\overline{6}$, hp 4, 12 bp windows

Multiplier	coderatecode	totstrandlen
0.5	3 (0.5)	210
1.0	4 (0.33)	182
1.5	4 (0.33)	182
2.0	4 (0.33)	182
2.5	4 (0.33)	182

(b) GC 40 - 60, hp 3, 10 bp windows

Table 1: Hedges parameters used for the rate analysis.

to increase the amount of redundancy available for error correction. In our fixed parameter evaluations, we used a sequence length of 254 bases, as this is the amount of code DNA characters per sequence, according to the Hedges authors. In contrast, for the cost evaluation, we decreased the number of bases per strand to 210, since this was the lowest value that led to a successful decoding without errors in the presence of literature error rates. We used the common constraints of a GC content between 40 % and 60 % in 10 base pair windows and a maximum homopolymer length of 3. The Python wrapper of Hedges requires that the decoding input is sorted by packet number and that the strands of each packet are also sorted; therefore, we evaluated all codes with ordered sequences. For other parameters, the default values were used in the evaluations. For the rate analysis, we adjusted the parameters (the sequence length and code rate) to achieve the successful decoding 100 out of 100 times for a given error rate with as few nucleotides as possible. The parameters are shown in Table 1

1.3 DNA Fountain

Installation

DNA Fountain [3] was downloaded from https://github.com/TeamErlich/dna-fountain, commit 4b2338b (the most recent as of April 12, 2022). A Python 2.7 virtualenv environment was created to install the packages as described by the INSTALL file in the repository linked above. The reedsolo version used was 0.3, since newer versions are incompatible with DNA Fountain.

Configuration

Based on the example for en- and decoding provided in the README file of DNA Fountain, we adjusted the GC content to be between 40 % and 60 %, and the maximum homopolymer length to 3. In our error correction performance evaluations, we used a chunk size of 13 with a maximum amount of fragments created at 2,000, leading to a total of 152,000 bases to encode the 4.8 KB input data. For the cost evaluations, we left the chunk size at 13 and decreased the maximum amount of fragments created to 1,500. Further decreasing the chunk size or the number of fragments generated led to a strong decrease in error correction performance, to the point of decoding failures in the absence of errors. Other parameters were kept in the default state as listed in the README file.

1.4 DNA-Aeon

Installation

DNA-Aeon was downloaded from https://github.com/MW55/DNA-Aeon. The installation was carried out as described in the README file.

Configuration

Using the generate_codebook.py script, we generated a codebook containing codewords with a GC content between 40 % and 60 % of 10 base pair lengths and with a maximum homopolymer length of 3. We did not exclude any motifs, since no other code evaluated supported undesired motifs in the implementations provided. In our error correction performance evaluations, we used a chunk size of 10, step size (the interval between synchronization markers) of 1, and a Raptor fountain overhead limit of 0.45. In the cost comparison, we used a chunk size of 20, a step size of 5, and a Raptor fountain overhead limit of 0.2.

2 *In-silico* Results

2.1 Substitution error rates evaluations

Errors	Bases	Success %	BER
1,000	83,421	100	0.012
1,500	83,421	94	0.018
2,000	83,421	0	0.024

(a) Grass code

Errors	Bases	Success %	BER
1,000	152,000	100	0.007
1,500	152,000	64	0.01
2,000	152,000	4	0.013
2,500	152,000	0	0.016

(b) DNA Fountain

Errors	Bases	Success %	BER
2,000	64,770	100	0.031
2,500	64,770	98	0.039
3,000	64,770	77	0.046
3,500	64,770	42	0.054
4,000	64,770	8	0.061
4,500	64,770	1	0.069
5,000	64,770	0	0.077

(c) Hedges

Errors	Bases	Success %	BER
4,500	64,635	100	0.070
5,000	64,635	95	0.077
5,500	64,635	0	0.085

(d) DNA-Aeon

2.2 Substitution, insertion, deletion error rate evaluations

Substitutions	Deletions	Insertions	Errors	Bases	Success %	BER
180	62	29	271	152,000	100	0.002
361	124	59	544	152,000	96	0.004
723	249	118	1,090	152,000	93	0.007
1,085	373	177	1,635	152,000	41	0.011
1,447	498	237	2,182	152,000	0	0.014

Table 2: DNA Fountain

Substitutions	Deletions	Insertions	Errors	Bases	Success %	BER
1,387	478	227	2,092	64,770	100	0.032
1,541	531	252	2,324	64,770	94	0.036
1,695	584	277	2,556	64,770	95	0.039
1,849	637	303	2,789	64,770	81	0.043
2,003	690	328	3,021	64,770	56	0.047
2,158	743	353	3,254	64,770	38	0.050
2,312	796	378	3,486	64,770	16	0.054
2,466	849	404	3,719	64,770	8	0.057
2,620	902	429	3,951	64,770	0	0.061

Table 3: Hedges

Substitutions	Deletions	Insertions	Errors	Bases	Success %	BER
2,768	954	453	4,175	64,635	100	0.065
2,922	1,007	478	4,407	64,635	99	0.068
3,076	1,060	504	4,640	64,635	77	0.072
3,230	1,113	529	4,872	64,635	4	0.075
3,384	1,166	554	5,104	64,635	0	0.079

Table 4: DNA-Aeon

2.3 Rate analysis

Substitutions	Deletions	Insertions	Errors	Bases	Multiplier	BER
438	151	71	657	36,844	0.5	0.018
999	344	163	1,506	41,978	1.0	0.036
1,875	646	307	2,828	52,542	1.5	0.054
3,634	1,252	595	5,481	76,349	2.0	0.072
5,425	1,869	889	8,183	91,182	2.5	0.09

Table 5: DNA-Aeon, GC $33.\overline{3}$ - $66.\overline{6}$, hp 4, 12 bp intervals

Substitutions	Deletions	Insertions	Errors	Bases	Multiplier	BER
534	184	87	805	44,880	0.5	0.018
1,068	368	175	1,611	44,880	1.0	0.036
1,911	658	313	2,882	53,550	1.5	0.054
4,418	1,522	723	6,663	92,820	2.0	0.072
5,522	1,902	904	8,328	92,820	2.5	0.09

Table 6: Hedges, GC $33.\overline{3}$ - $66.\overline{6}$ %, hp 4, 12 bp windows

Substitutions	Deletions	Insertions	Errors	Bases	Multiplier	BER
462	159	75	696	38,880	0.5	0.018
1,092	376	179	1,647	45,900	1.0	0.036
2,144	738	351	3,233	60,078	1.5	0.054
3,727	1,284	610	5,621	78,306	2.0	0.072
5,516	1,900	904	8,320	92,721	2.5	0.09

Table 7: DNA-Aeon, GC 40 - 60 %, hp 3, 10 bp intervals

Substitutions	Deletions	Insertions	Errors	Bases	Multiplier	BER
637	219	104	960	53,550	0.5	0.018
2,209	761	361	3,331	92,820	1.0	0.036
3,313	1,141	542	4,996	92,820	1.5	0.054
4,418	1,522	723	6,663	92,820	2.0	0.072
5,522	1,902	904	8,328	92,820	2.5	0.09

Table 8: Hedges, GC 40 - 60 %, hp 3, 10 bp windows

3 mCGR Evaluation

To analyze the fragment distribution in the encoded data, we used matrix chaos game representation (mCGR) [4] and the R package kaos [5]. mCGR enables the mapping of a sequence to a unique matrix element. The method is an extension of chaos game representation (CGR). While the original CGR is applied to map one sequence, the mCGR can be considered as a $2^k \times 2^k$ matrix, representing all possible sequences of the length k. Different constraints will emerge as different fractal patterns in the mCGR, as shown for the GC content, homopolymers, and hamming distance in [6] It is therefore possible to use mCGR to construct codewords adhering to user-defined constraints. Since the mCGR allows visual identification of constraints, we adapted it for code evaluation in terms of constraint adherence and overrepresented subsequence identification.

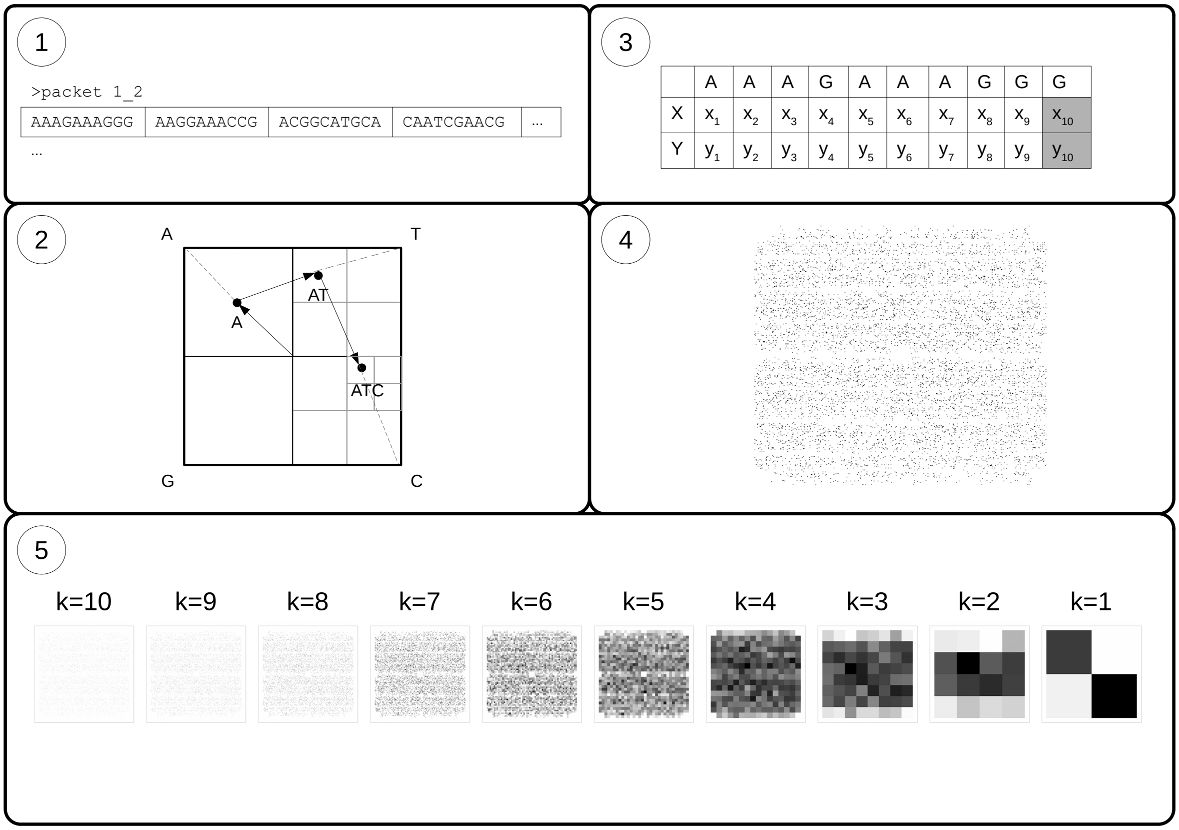

Figure 1: mCGR approach for code-word evaluation: the encoded data is split (1), followed by the application of the CGR algorithm (2). (3) shows the representation of a 10 nucleotide long sequence by 10 x- and y-coordinates. (4) represents the combination of the results from (3) into a single CGR. In (5), the subsequences are clustered by decreasing the resolution of the mCGR.

Figure 1 shows the mCGR based approach for code-word evaluation. In the first step (Figure 1 (1)), we split the encoded files, namely a 4.8 KB text file containing the fairy tale Dornroschen, encoded using Hedges, DNA Fountain, DNA-Aeon, and Grass et al. into fragments of length 10. In (2), the CGR algorithm of the R Package kaos [5] was applied for each of the fragments. This algorithm assigns the four nucleotides of DNA to the edges of a square starting at the middle. For the first nucleotide in a given input sequence (in our case, a ten nt fragment), a point P_1 s calculated, half the distance to the edge with the corresponding nucleotide. For P_2, half the distance to the edge with the second nucleotide from P_1 is calculated. This recursion is repeated for the complete sequence. A sequence of 10 nt can the represented by 10 x- and y-coordinates (3). Due to the recursive nature of the algorithm, the complete input sequence can be retrieved from the last coordinate. We decided to use a length of 10 for multiple reasons. The underlying codebook for DNA-Aeon was constructed with words of the length of 10. Thus, this will allow us to investigate the GC content constraint for fragments of the length of 10. For calculating the last coordinates for each packet, the mCGR would have been very sparse and would result in a matrix of $2^n \times 2^n$, with n being the sequence lengths. In contrast, the fragmentation of the sequences enables better visualization and provides more information on the distribution of particular patterns within the code. In the next step (Figure 1 (4)) , the results from (3) are combined into a single CGR. Within this

representation, each point represents one fragment. This CGR can be used to calculate the so-called frequency chaos game representation (FCGR). To construct the FCGR, the CGR is divided into a grid of size $2^n x 2^n$. For our purposes, a $2^{10} \times 2^{10}$ grid. The resulting matrix represents all possible words of length 10 (mCGR). Each element in the matrix represents a sequence. Therefore it allows the identification of homopolymers, motifs, and highly frequent fragments or subsequences, respectively. Additionally, we decreased the grid size for $2^k \times 2^k$ with $k = 1..10$. Due to the nature of CGR, this will cluster fragments with the same postfixes together. The results for k=10 are shown in the first subfigure of Figure 2 to Figure 5. We then decreased the grid size of the matrix to a smaller k. For $k = 1$, the sequences with the same postfix of length k are clustered together (Figure 2 to Figure 5, last subfigure). For k=2, the sequences where the two last nucleotides are equal are clustered together, and so on. The number of sequences is represented as one square in different gray values, while white corresponds to low-frequency and black to high-frequency.

For all four codes, at large k, very sparse patterns can be observed. In most cases, there are only a few small light-gray squares on a white background. For k=10, the matrix is a matrix of 1,024 x 1,024 elements. Decreasing k, and therefore the clustering of similar fragments, allows better visualization and interpretation of the data. Figure 2 shows the results of our mCGR analysis of the Grass code. For decreasing k, a cross-like pattern emerges. This means that the construction of the code follows a symmetric pattern for the selection of the nucleotides. The corners of the mCGR represent homopolymers. For instance, for k=4, the corners are white, because homopolymers of a length of 4 are excluded. For k=5, the four middle elements and the middle elements at the borders of the matrix remain empty. Those elements represent the words starting with a homopolymer of a length 4.

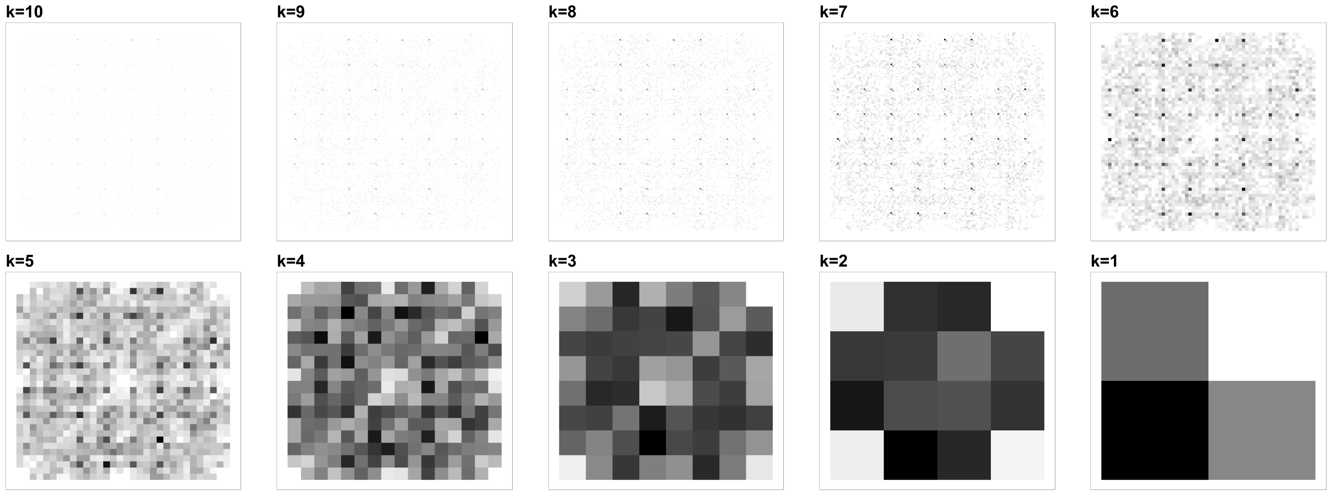

Figure 2: Grass

In Figure 3, the same pattern for the homopolymer constrain can be found. For k=4, the corners are white, and for k=5, the middle elements in the matrix and the borders remain white. Additionally, a chess-like pattern can be observed in all sizes of k. The pattern starts at k=2, where AA, AT, CA, CT, AG, AC, CG, and CC, were over-represented compared to the others. Because of the fractal arrangement, this pattern continues as a fractal chessboard. The fragments containing these nucleotides are darker in the mCGRs for higher k.

Figure 4 shows the results of the mCGR analyzes for the Hedges code. Three distinctive clusters can be found in the mCGR. For k=3, the clusters represent the elements GTA, TGC, and GTC. For k=4, the same pattern exists, while the cluster for TGC separates into a cluster of four elements. These elements represent the sequences NTGC (N for any). The other two clusters remain as a single element in the matrices with higher resolution, indicating a longer motif over-represented compared to the other motifs in the sequence.

In Figure 5 the results for DNA-Aeon are shown. For k=2, the elements in the second and third-row are over-represented compared to those in the first and fourth. The mCGR is arranged as AT-GC. To this end, the 2nd and 3rd row have a 50% GC-Content in the last two nucleotides. The homopolymers constrained can be observed at k=5 and k=4. The distribution appears random, and no visible pattern can be observed.

Comparing the mCGR analysis of all four codes, DNA-Aeon is the only code with patterns emerging only from the constraints. The other three codes have a characteristic pattern. We have chosen this approach to analyze if the code space is fully exploited. All mCGR show the encoding of the same file Dornroeschen, so the patterns may be partially connected to the encoded data. However, the approach indicates that the mCGR analysis might be a possible method to identify the applied encoding in case this information is lost. Additionally, the absence of patterns in DNA-Aeon may be useful for cryptography. Since DNA-Aeon encodes data based on a codebook built with mCGR, it would be possible to encode meta-information in the mCGR.

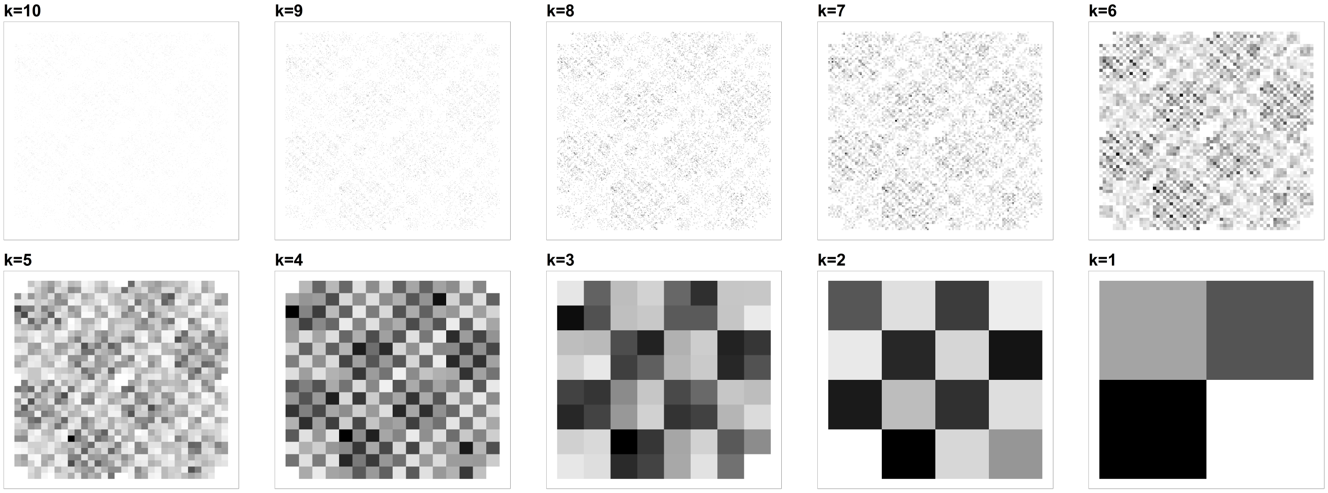

Figure 3: DNA Fountain

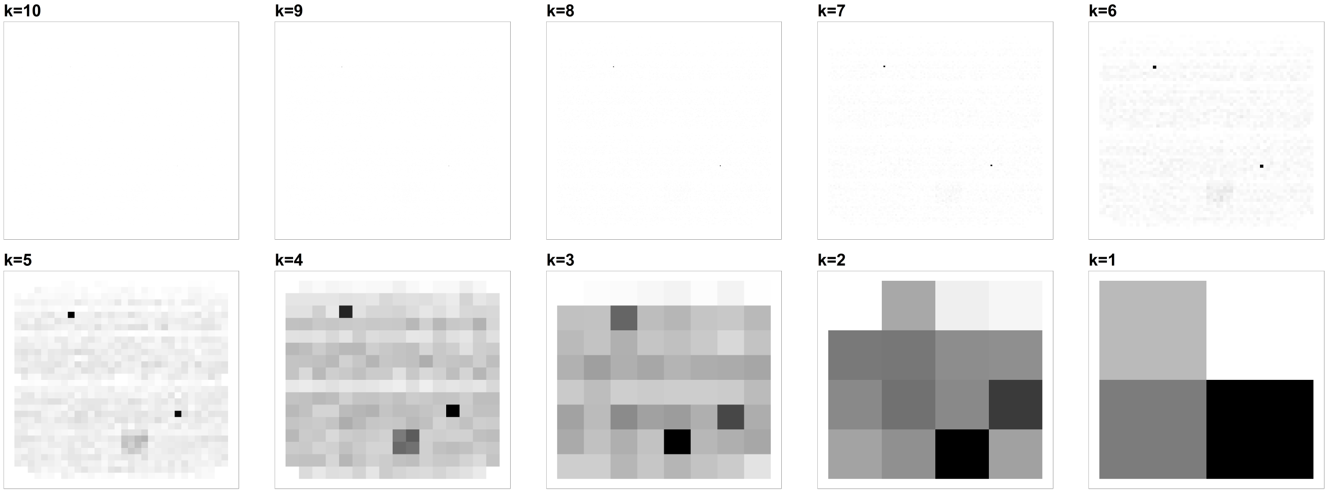

Figure 4: Hedges

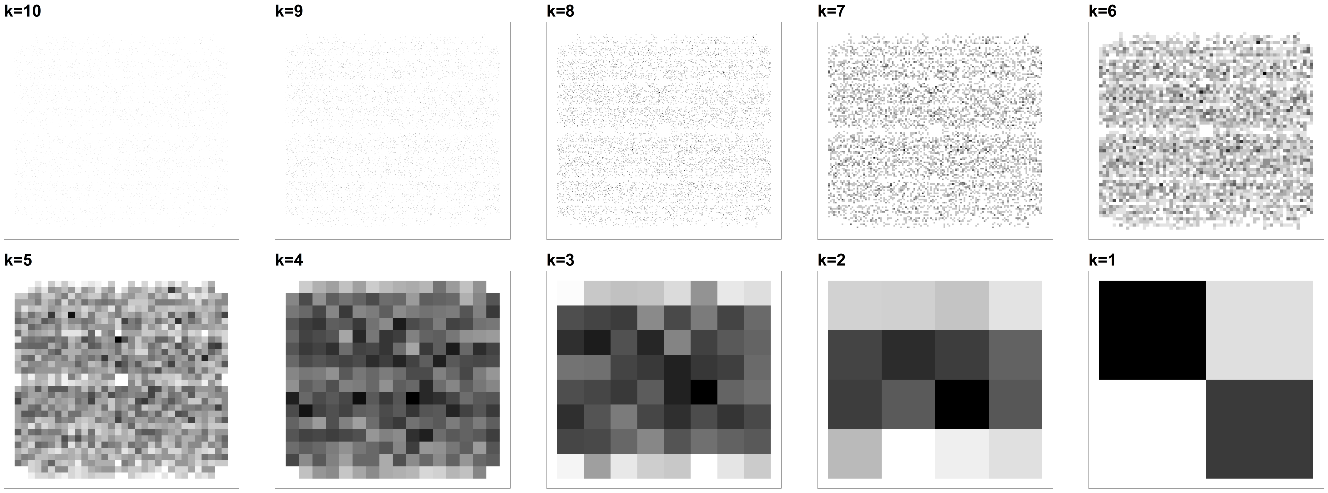

Figure 5: DNA Aeon

4 *In-vitro* Analysis

4.1 Encoded files

We encoded a 29.9 KB PNG file of the MOSLA reseach cluster logo (Figure 6) and a 47.1 KB JPEG file of the Enterprise NCC 1701-D.

Figure 6: MOLSA logo: logo of the MOSLA research cluster that was encoded for the *in-vitro* analysis.

We furthermore encoded a 4.8 KB raw text file of the German fairy tale Dornröschen (sleeping beauty). The German text version we used was as follows:

Ein Koenig und eine Koenigin kriegten gar keine Kinder, und haetten so gern eins gehabt. Einmal sass die Koenigin im Bade, da kroch ein Krebs aus dem Wasser ans Land und sprach: 'dein Wunsch wird bald erfuellt werden und du wirst eine Tochter zur Welt bringen.' Das traf auch ein, und der Koenig war so erfreut ueber die Geburt der Prinzessin, dass er ein grosses Fest anstellen liess, und dazu lud er auch die Feen ein, die im Lande waren, weil er nur zwoelf goldene Teller hatte, konnte er eine nicht einladen: es waren ihrer nemlich dreizehen. Die Feen kamen zu dem Fest, und beschenkten das Kind am Ende desselben: die eine mit Tugend, die zweite mit Schoenheit und so die andern mit allem, was nur auf der Welt herrlich und zu wuenschen war, wie aber eben die elfte ihr Geschenk gesagt hatte, trat die dreizehnte herein, recht zornig, dass sie nicht war eingeladen worden und rief: 'weil ihr mich nicht gebeten, so sage ich euch, dass eure Tochter in ihrem funfzehnten Jahre an einer Spindel sich stechen und todt hinfallen wird.' Die Eltern erschracken, aber die zwoelfte Fee hatte noch einen Wunsch zu thun, da sprach sie: 'es soll aber kein Tod seyn, sie soll nur hundert Jahr in einen tiefen Schlaf fallen.' Der Koenig hoffte immer noch sein liebes Kind zu erretten, und liess den Befehl ausgehen, dass alle Spindeln im ganzen Koenigreich sollten abgeschafft werden. Die Prinzessin aber wuchs heran, und war ein Wunder von Schoenheit. Eines Tags, als sie ihr funfzehntes Jahr eben erreicht hatte, war der Koenig und die Koenigin ausgegangen, und sie ganz allein im Schloss, da ging sie aller Orten herum nach ihrer Lust, endlich kam sie auch an einen alten Thurm. Eine enge Treppe fuehrte dazu, und da sie neugierig war, stieg sie hinauf und gelangte zu einer kleinen Thuere, darin steckte ein gelber Schluessel, den drehte sie um, da sprang die Thuere auf und sie war in einem kleinen Stuebchen, darin sass eine alte Frau und spann ihren Flachs. Die alte Frau gefiel ihr wohl, und sie machte Scherz mit ihr und sagte, sie wollte auch einmal spinnen, und nahm ihr die Spindel aus der Hand. Kaum aber hatte sie die Spindel angeruehrt, so stach sie sich damit, und alsbald fiel sie nieder in einen tiefen Schlaf. In dem Augenblick kam der Koenig mit dem ganzen Hofstaat zurueck, und da fing alles an einzuschlafen, die Pferde in den Staellen, die Tauben auf dem Dach, die Hunde im Hof, die Fliegen an den Waenden, ja das Feuer, das auf dem Heerde flackerte, ward still und schlief ein, und der Braten hoerte auf zu brutzeln, und der Koch liess den Kuechenjungen los, den er an den Haaren ziehen wollte, und die Magd liess das Huhn fallen, das sie rupfte und schlief, und um das ganze Schloss zog sich eine Dornhecke hoch und immer hoeher, so dass man gar nichts mehr davon sah. Prinzen, die von dem schoenen Dornroeschen gehoert hatten, kamen und wollten es befreien, aber sie konnten durch die Hecke nicht hindurch dringen, es war als hielten sich die Dornen fest wie an Haenden zusammen, und sie blieben darin haengen und kamen jaemmerlich um. So waehrte das lange, lange Jahre: da zog einmal ein Koenigssohn durch das Land, dem erzaehlte ein alter Mann davon, man glaube, dass hinter der Dornhecke ein Schloss stehe, und eine wunderschoene Prinzessin schlafe darin mit ihrem ganzen Hofstaat; sein Grossvater habe ihm gesagt, dass sonst viele Prinzen gekommen waeren und haetten hindurchdringen wollen, sie waeren aber in den Dornen haengen geblieben und todtgestochen worden. 'Das soll mich nicht schrecken, sagte der Koenigssohn, ich will durch die Hecke dringen und das schoene Dornroeschen befreien;' da ging er fort, und wie er zu der Dornhecke kam, waren es lauter Blumen, die thaten sich von einander, und er ging hindurch, und hinter ihm wurden es wieder Dornen. Da kam er ins Schloss, und in dem Hof lagen die Pferde und schliefen, und die bunten Jagdhunde, und auf dem Dach sassen die Tauben und hatten ihre Koepfchen in den Fluegel gesteckt, und wie er hineinkam, schliefen die Fliegen an den Waenden, und das Feuer in der Kueche, der Koch und die Magd, da ging er weiter, da lag der ganze Hofstaat und schlief, und noch weiter, der Koenig und die Koenigin; und es war so still, dass einer seinen Athem hoerte, da kam er endlich in den alten Thurm, da lag

Dornroeschen und schlief. Da war der Koenigssohn so erstaunt ueber ihre Schoenheit, dass er sich bueckte und sie kuesste, und in dem Augenblick wachte sie auf, und der Koenig und die Koenigin, und der ganze Hofstaat, und die Pferde und die Hunde, und die Tauben auf dem Dach, und die Fliegen an den Waenden, und das Feuer stand auf und flackerte und kochte das Essen fertig, und der Braten brutzelte fort, und der Koch gab dem Kuechenjungen eine Ohrfeige, und die Magd rupfte das Huhn fertig. Da ward die Hochzeit von dem Koenigssohn mit Dornroeschen gefeiert, und sie lebten vergnuegt bis an ihr Ende.

4.2 Encoding parameters

A codebook was generated that restricts the GC content of the encoded data to 40 % - 60 %, no homopolymers longer than three, and does not allow the occurrence of the motifs shown in Table 9, since these were used in the biological processing steps.

Description	Sequence
Universal Multiplex Adapter F	CTCGTAGACTGCGTACCA
Universal Multiplex Adapter R	GACGATGAGTCCTGAGTA
Nextera Kit adapter	CTGTCTCTTATACACATCT
Nextera Kit adapter	TCGTCGGCAGCGTCAGATGTGTATAAGAGACAG
Nextera Kit adapter	GTCTCGTGGGCTCGGAGATGTGTATAAGAGACAG

Table 9: Motifs that were excluded from the encoding space, as they were used for biological processing.

The DNA-Aeon parameters used to encode the data is shown in Table 10.

File	Chunk size	step size	Overhead limit	Encoded sequences	Bases per sequence
Dornröschen, CRC interval 1	16	1	0.4	429	156
Dornröschen, CRC interval 3	25	3	0.4	276	156
Dornröschen, CRC interval 5	28	5	0.4	247	156
MOSLA logo, CRC interval 2	22	2	0.4	1,951	156
Enterprise, CRC interval 3	25	3	0.4	2,704	156

Table 10: Encoding parameters for the files used in the *in-vitro* evaluations.

4.3 Synthesis, amplification, and sequencing

Information carrying 156 nucleotide long DNA sequences were flanked from both sides by 20 nucleotide long adapter sequences, previously published as random-access-compatible indexes for large-scale DNA-based storage [7]. Each encoded file library received a unique pair of indexes, which enable access to a specific library from a pool by PCR amplification using the compatible primer pair (Table 11). The resulting 196 nucleotide long sequence libraries were de novo synthesized by Twist Bioscience as a single-stranded DNA oligonucleotide pool. The samples were resuspended upon arrival in 10 mM Tris-HCl, pH 8.0, to the concentration of 10 ng/µL and stored at –80° C. The single library was amplified in a low cycle PCR using Q5 polymerase (NEB) with subsequent amplification product visualization on two percent agarose gel electrophoresis and the product clean up using E.Z.N.A.® Cycle Pure Kit (Omega Bio-tek, Inc.) (Figure 7). All used primers are listed in Table 12. The amplified libraries were used as templates for the second low cycle PCR reaction with primers adding 5' transposase adapters (Tn5 transposase, TDE 15027865, Illumina) was performed in replacement of the tagmentation step in Nextera XT DNA Library Prep (Illumina), followed by the manufacturer specified protocol (Figure 7). The quality control of the libraries prior to sequencing included DNA quantification via iT dsDNA High-Sensitivity Assay Kit (Invitrogen) on Qubit 4 Fluorometer (Thermo Fisher), indexed libraries quantification using KAPA Library Quantification Kit (Roche), and fragment size estimation using HS NGS Fragment 1-6000bp kit on the Fragment Analyzer 5200 (Agilent Technologies). The sequencing was carried

Library	Number of sequences	5’ adaptersequence 5’ → 3’	3’ adaptersequence 5’ → 3’	Organick et al., 2018, primer pair #
Enterprise	2704	AAGGCAAGTTGTTACCAGCA	TTGGTTTGATTACGGTCGCA	1
MOSLA logo	1951	AATGTCGAAGAAAGCCGGTT	AATTCGGTTTGAAGCACGGT	15
Dornroeschen_247 (CRC5)	247	TCCATTGCGTCAACCGTTAT	TCACGAAACTTCCATTGGCA	9
Dornroeschen_276 (CRC3)	276	TACCGCATCCTTATTCGAGC	TCTGGTGCAAGCCAATGAAA	30
Dornroeschen_429 (CRC1)	429	TCCTGCTTGCGTTAAATGGA	TTCCGCAAGACTTATTGGCA	19

Table 11: Oligonucleotide libraries varied by the number of unique sequences. Each unique sequence in the library was flanked by adapter sequences at the 5’ and 3’ ends.

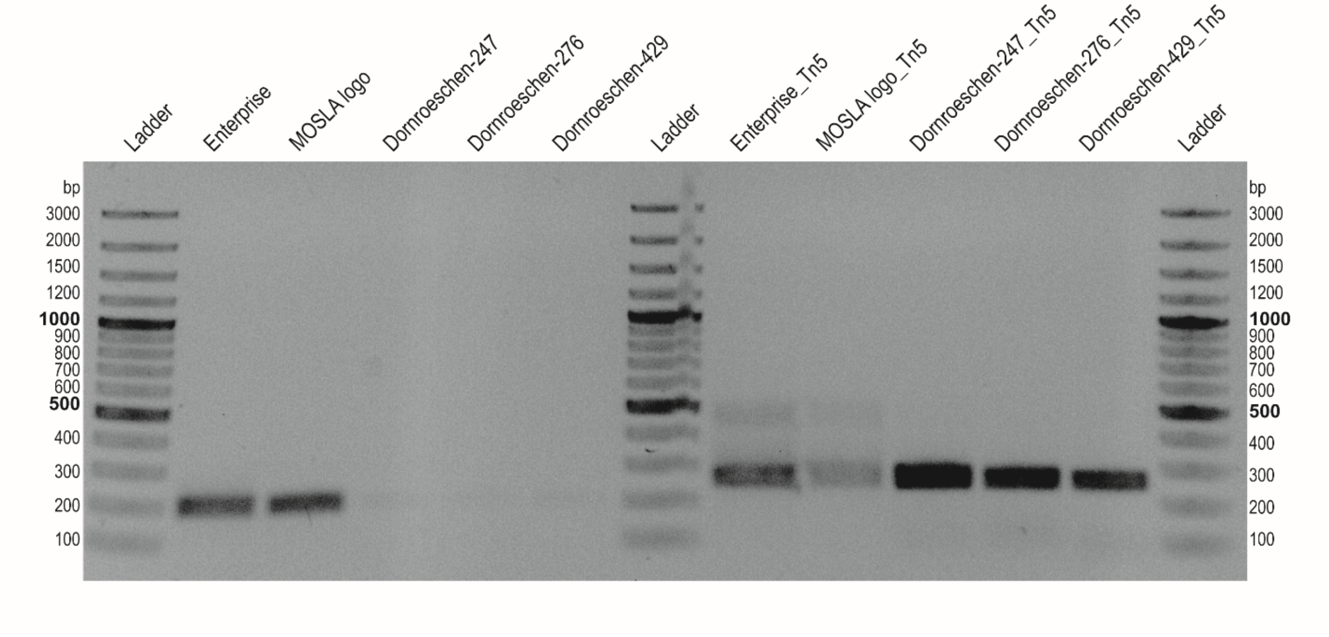

Figure 7: PCR amplification product visualization on two % agarose gel. The oligonucleotide libraries were amplified from the pool in the first PCR reaction. The PCR product from the first reaction was used in the second reaction to add Tn5 adapters.Ladder - Thermo Scientific™ O’GeneRuler™ 100 bp Plus DNA Ladder. The experiment was carried out without repetitions.

out with a 20 % PhiX control library on MiSeq v3 PE300 platform. The processing, pairing, and mapping of the raw sequencing data was carried out using Geneious Prime® 2022.0.1. Table 13 summarizes the sequencing output per oligonucleotide library.

4.4 Raw read processing

To process the raw reads, we used the sequence processing pipeline Natrix [8] up until the dereplication of the dataset, after which we either used additional 97 % similarity clustering or no clustering. The DAG of an example dataset is shown in Figure 8.

4.5 Decoding

Besides stopping the Natrix pipeline after the dereplication of the data set, we also evaluated the ability of DNA-Aeon to decode the input data using an additional 97 % similarity clustering. After the initial successful decoding of the datasets using 100 % of the raw FASTQ data, we gradually decreased the number of reads in the raw FASTQ files, up until the point DNA-Aeon was not able to decode the data anymore. The lowest percentage of raw reads used that still allowed the successful decoding of the input data, without similarity clustering, is shown in Table 14, and including the similarity clustering in Table 15.

Primer name	Primer sequence 5' → 3'
Enterprise_Fw	AAGGCAAGTTGTTACCAGCA
Enterprise_Rv	TGCGACCGTAATCAAACCAA
Enterprise-Tn5_Fw	tcgtcggcagcgtcagatgtgtataagagacagAAGGCAAGTTGTTACCAGCA
Enterprise-Tn5_Rv	gtctcgtgggctcggagatgtgtataagagacagTGCGACCGTAATCAAACCAA
MOSLA logo_Fw	AATGTCGAAGAAAGCCGGTT
MOSLA logo_Rv	ACCGTGCTTCAAACCGAATT
MOSLA logo-Tn5_Fw	tcgtcggcagcgtcagatgtgtataagagacagAATGTCGAAGAAAGCCGGTT
MOSLA logo-Tn5_Rv	gtctcgtgggctcggagatgtgtataagagacagACCGTGCTTCAAACCGAATT
Dornroeschen_247_Fw	TCCATTGCGTCAACCGTTAT
Dornroeschen_247_Rv	TGCCAATGGAAGTTTCGTGA
Dornroeschen-Tn5_247_Fw	tcgtcggcagcgtcagatgtgtataagagacagTCCATTGCGTCAACCGTTAT
Dornroeschen-Tn5_247_Rv	gtctcgtgggctcggagatgtgtataagagacagTGCCAATGGAAGTTTCGTGA
Dornroeschen_276_Fw	TACCGCATCCTTATTCGAGC
Dornroeschen_276_Rv	TTTCATTGGCTTGCACCAGA
Dornroeschen-Tn5_276_Fw	tcgtcggcagcgtcagatgtgtataagagacagTACCGCATCCTTATTCGAGC
Dornroeschen-Tn5_276_Rv	gtctcgtgggctcggagatgtgtataagagacagTTTCATTGGCTTGCACCAGA
Dornroeschen_429_Fw	TCCTGCTTGCGTTAAATGGA
Dornroeschen_429_Rv	TGCCAATAAGTCTTGCGGAA
Dornroeschen-Tn5_429_Fw	tcgtcggcagcgtcagatgtgtataagagacagTCCTGCTTGCGTTAAATGGA
Dornroeschen-Tn5_429_Rv	gtctcgtgggctcggagatgtgtataagagacagTGCCAATAAGTCTTGCGGAA

Table 12: Primers used for PCR-based library amplification and addition of Tn5 transposase adapters.

Library	Number of sequenced nucleotides per library	Number of reads per library	Number of mapped reads per sequence
Enterprise	1,312,842,804	4,361,604	33-6,493
MOSLA logo	1,220,708,510	4,055,510	9-8,692
Dornroeschen_247	1,162,093,576	3,860,776	3,370-29,886
Dornroeschen_276	693,496,174	2,303,974	168-19,002
Dornroeschen_429	1,401,176,672	4,655,072	999-25,767

Table 13: Summary of the sequencing output per library. While the estimated target output per library was four million reads, there is a variation in the generated data, as indicated by the number of reads per library.

Input file	pq	mq	raw multiple of encoded	processed multiple of encoded	raw sequence numbers	percentage of raw reads used
Dornröschen, CRC interval 1	0.3	10	48.83	1.284	20,948	0.9
	0.3	20	54.254	1.324	23,275	1.0
	0.3	30	108.51	1.275	46,551	2.0
	0.6	10	48.83	1.277	20,948	0.9
	0.6	20	54.254	1.324	23,275	1.0
	0.6	30	108.51	1.275	46,551	2.0
	0.9	10	108.51	1.2	46,551	2.0
	0.9	20	108.51	1.179	46,551	2.0
	0.9	30	271.275	1.392	116,377	5.0
Dornröschen, CRC interval 3	0.3	10	41.739	1.239	11,520	1.0
	0.3	20	41.739	1.225	11,520	1.0
	0.3	30	83.478	1.178	23,040	2.0
	0.6	10	41.739	1.232	11,520	1.0
	0.6	20	41.739	1.217	11,520	1.0
	0.6	30	83.478	1.174	23,040	2.0
	0.9	10	208.692	1.967	57,599	5.0
	0.9	20	208.692	1.96	57,599	5.0
	0.9	30	208.692	1.236	57,599	5.0
Dornröschen, CRC interval 5	0.3	10	54.709	1.34	13,513	0.7
	0.3	20	54.709	1.316	13,513	0.7
	0.3	30	156.308	1.36	38,608	2.0
	0.6	10	54.709	1.328	13,513	0.7
	0.6	20	54.709	1.304	13,513	0.7
	0.6	30	156.308	1.36	38,608	2.0
	0.9	10	156.308	1.462	38,608	2.0
	0.9	20	156.308	1.433	38,608	2.0
	0.9	30	390.765	1.49	96,519	5.0
Enterprise, CRC interval 3	0.3	10	80.651	1.738	218,080	10.0
	0.3	20	80.651	1.723	218,080	10.0
	0.3	30	80.651	1.104	218,080	10.0
	0.6	10	80.651	1.726	218,080	10.0
	0.6	20	80.651	1.713	218,080	10.0
	0.6	30	80.651	1.1	218,080	10.0
	0.9	10	161.302	1.516	436,160	20.0
	0.9	20	161.302	1.508	436,160	20.0
	0.9	30	241.953	1.266	654,241	30.0
MOSLA logo, CRC interval 2	0.3	10	51.967	1.235	101,388	5.0
	0.3	20	51.967	1.222	101,388	5.0
	0.3	30	103.934	1.297	202,776	10.0
	0.6	10	51.967	1.23	101,388	5.0
	0.6	20	51.967	1.22	101,388	5.0
	0.6	30	103.934	1.291	202,776	10.0
	0.9	10	207.868	1.734	405,551	20.0
	0.9	20	207.868	1.721	405,551	20.0
	0.9	30	207.868	1.141	405,551	20.0

Table 14: Metadata of the lowest amount of reads needed to successfully decode the input data for different quality and assembly thresholds. Mq = the mean minimal quality of reads to not be discarded, pq = minimal quality of the read assembly to not be discarded, raw multiple of encoded: the number of raw reads, as a multiple of the number of encoded sequences, required for successful decoding, processed multiple of encoded = the minimal amount of assembled reads after processing that was required for successful decoding, raw sequence numbers = the total amount of sequences before processing needed for decoding, percentage of raw reads used = the percentage of reads from the initial FASTQ file that was used for processing and decoding.

Input file	pq	mq	raw multiple of encoded	processed multiple of encoded	raw sequence numbers	percentage of raw reads used
Dornröschen, CRC interval 1	0.3	10	48.83	0.809	20,948	0.9
	0.3	20	54.254	0.841	23,275	1.0
	0.3	30	108.51	0.804	46,551	2.0
	0.6	10	48.83	0.809	20,948	0.9
	0.6	20	54.254	0.839	23,275	1.0
	0.6	30	108.51	0.804	46,551	2.0
	0.9	10	108.51	0.802	46,551	2.0
	0.9	20	108.51	0.79	46,551	2.0
	0.9	30	271.275	0.814	116,377	5.0
Dornröschen, CRC interval 3	0.3	10	41.739	0.812	11,520	1.0
	0.3	20	41.739	0.804	11,520	1.0
	0.3	30	83.478	0.761	23,040	2.0
	0.6	10	41.739	0.812	11,520	1.0
	0.6	20	41.739	0.804	11,520	1.0
	0.6	30	83.478	0.761	23,040	2.0
	0.9	10	208.692	0.964	57,599	5.0
	0.9	20	208.692	0.96	57,599	5.0
	0.9	30	208.692	0.768	57,599	5.0
Dornröschen, CRC interval 5	0.3	10	54.709	0.85	13,513	0.7
	0.3	20	54.709	0.834	13,513	0.7
	0.3	30	156.308	0.806	38,608	2.0
	0.6	10	54.709	0.842	13,513	0.7
	0.6	20	54.709	0.834	13,513	0.7
	0.6	30	156.308	0.806	38,608	2.0
	0.9	10	156.308	0.87	38,608	2.0
	0.9	20	156.308	0.854	38,608	2.0
	0.9	30	390.765	0.83	96,519	5.0
Enterprise, CRC interval 3	0.3	10	80.651	0.943	218,080	10.0
	0.3	20	80.651	0.935	218,080	10.0
	0.3	30	80.651	0.742	218,080	10.0
	0.6	10	80.651	0.938	218,080	10.0
	0.6	20	80.651	0.931	218,080	10.0
	0.6	30	80.651	0.741	218,080	10.0
	0.9	10	161.302	0.876	436,160	20.0
	0.9	20	161.302	0.87	436,160	20.0
	0.9	30	241.953	0.783	654,241	30.0
MOSLA logo, CRC interval 2	0.3	10	51.967	0.784	101,388	5.0
	0.3	20	103.934	0.966	202,776	10.0
	0.3	30	103.934	0.789	202,776	10.0
	0.6	10	51.967	0.783	101,388	5.0
	0.6	20	103.934	0.964	202,776	10.0
	0.6	30	103.934	0.788	202,776	10.0
	0.9	10	207.868	0.916	405,551	20.0
	0.9	20	207.868	0.91	405,551	20.0
	0.9	30	207.868	0.738	405,551	20.0

Table 15: Metadata of the lowest amount of reads needed to successfully decode the input data for different quality thresholds and assembly thresholds, using a 97 % similarity clustering as the final processing step. Mq = mean minimal quality of reads to not be discarded, pq = minimal quality of the read assembly to not be discarded, raw multiple of encoded: the number of raw reads, as a multiple of the number of encoded sequences, required for successful decoding, processed multiple of encoded = the minimal amount of assembled reads after processing that was required for successful decoding, raw sequence numbers = the total amount of sequences before processing needed for decoding, percentage of raw reads used = the percentage of reads from the initial FASTQ file that was used for processing and decoding

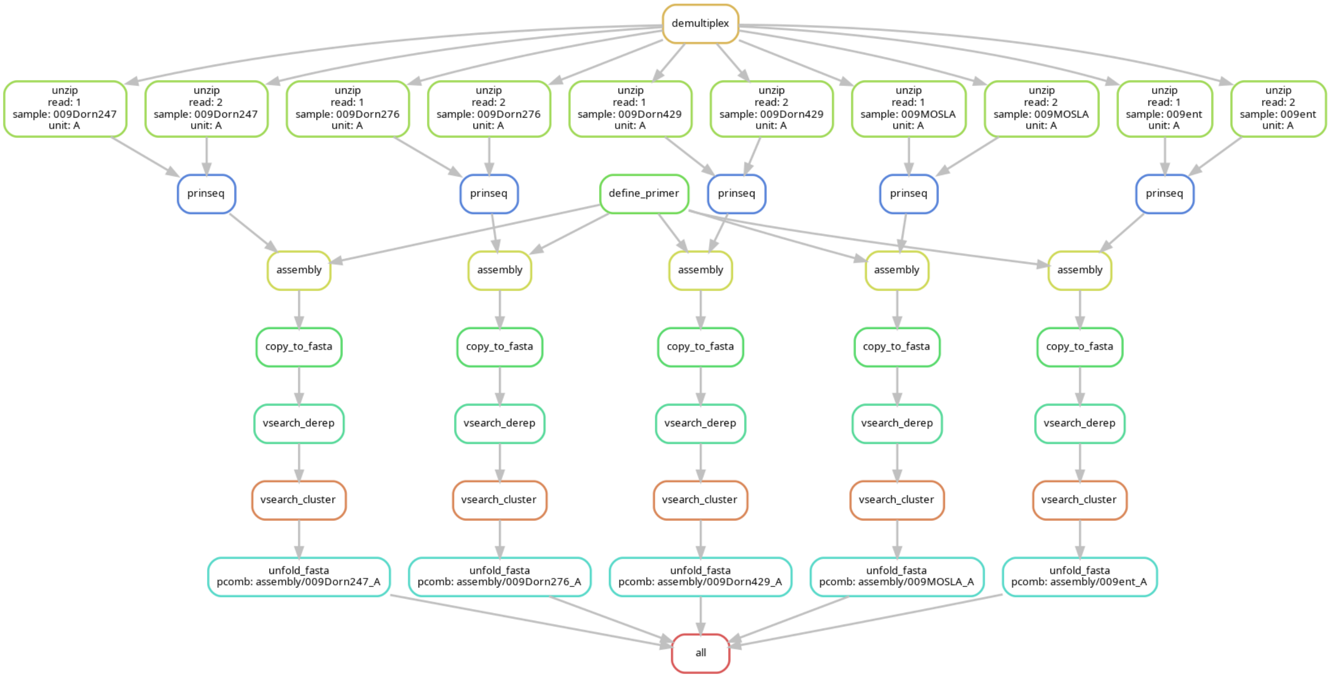

Figure 8: DAG of the step taken for the read processing, here shown with similarity clustering.

5 Mathematical description of DNA-Aeon

5.1 Outer encoding

The outer code of DNA-Aeon is a Raptor fountain code [9, 10]. It enables splitting the input file into a number of chunks $A = (a_1, a_2, ..., a_N)$ of length k, with an optional checksum and padding if the file size is not a multiple of k. An optional header chunk can be added to the start of the file including meta-information, such as the filename, the number of padding bytes at the end of the file as well as a file-wide checksum. Raptor fountain codes also require a degree distribution $\Omega(x)$, in our case, on $\mathbb{F}_2^k$. Each chunk can be represented as a vector $H = (h_1, h_2, ..., h_k)$ in $\mathbb{F}_2^k$. Raptor fountain codes use pre-coding of the chunks A into $\tilde{A} = (\tilde{a}_1, \tilde{a}_2, ..., \tilde{a}_V)$, with $V > N$, to allow the decoding of the N symbols of A in linear time with little overhead [9]. The V intermediate symbols are generated as described by Luby et al. [10], using a generator matrix G. This matrix is a $V \times N$ matrix over $\mathbb{F}_2$. Using the V intermediate symbols of length k each, the generation of a new packet f in the Raptor code can be described as follows. First, a unique seed s_f is chosen at random or in an incremental manner. This seed will then be used to initialize the pseudo random number generator, in our case a Mersenne-Twister, with which a degree distribution $\Omega(x)$ (known to both the en- and decoder) is sampled to retrieve the degree d_f of the packet. By using d_f as a weight, we can choose a vector $v_f = (v_{f1}, v_{f2}, \ldots, v_{fV})$ uniformly at random from $\mathbb{F}_2^V$, using the same seed (s_f) for the pseudo random number generator, such that the number of ones in v_f equals d_f. Thus, the resulting packet is of degree d_f. The output symbol δ_f will be created using:

$$\delta_f = \sum_{f=1}^{f=V} v_f \tilde{a}_f \tag{1}$$

Finally, the new packet p_f is a concatenation of the seed s_f and the output symbol δ_f: $p_f = (s_f, \delta_f)$. Optionally, one can choose to store the number of chunks N in the packet:

$$p_f = (N, s_f, \delta_f) \tag{2}$$

5.2 Symbol probabilities of the inner code

For a given source alphabet Σ of size S, $\Sigma = \{m_1, m_2, ..., m_S\}$, in our case $\Sigma = \{A, T, C, G\}$, where each symbol maps to an integer q as the symbol index in the range $\{0, 1, 2, ..., S-1\}$, the probability that a base b is inserted at position j by the encoder is given by the frequency of that base at this position in the set of codewords of length J that comprise the codebook $W = \{w_1, w_2, ..., w_L\}$:

$$\hat{P}(b_j) = \frac{1}{L} \sum_{i=1}^{L} \mathbf{1}_{\tilde{b}} \left(w_{ij} \right) \tag{3}$$

with

$$\tilde{b} = \{b\} \tag{4}$$

and

$$\mathbf{1}_{\tilde{b}}(w_{ij}) \coloneqq \begin{cases} 1 & \text{if } w_{ij} \in \tilde{b} \\ 0 & \text{if } w_{ij} \notin \tilde{b} \end{cases} \tag{5}$$

If the output of the encoder, or the input of the decoder, reaches a multiple of the codeword length J, a new codebook $\tilde{W}$ is generated, which is the difference of W and a set of forbidden prefixes F, that would lead to a constraint violation if incorporated into the DNA sequence:

$$\tilde{W} = W \setminus F \tag{6}$$

which is subsequently used for the probability calculations as our codebook. To simplify the description, we use the definition of the source symbol probabilities [11]:

$$P(z) = P(q = z), z = 0, ..., S-1 \tag{7}$$

These probabilities can then be used to define the cumulative probability distribution in the following way:

$$P_q(z) = \sum_{k=0}^{z} P(k), z = 0, ..., S-1 \tag{8}$$

5.3 Inner encoding

The inner encoder uses as its input a sequence R of N bytes $R = (r_1, r_2, ..., r_N)$, returned by the outer encoder, and inserts every τ bytes, with τ being the user-defined step-size parameter, an 8-Bit CRC checksum of the previous τ bytes, i.e., if $n \mod \tau = 0$:

$$\mathrm{CRC}(r_{n-\tau+1}, ..., r_n) \tag{9}$$

The resulting byte sequence including the CRCs is denoted as $\tilde{R} = (\tilde{r_1}, \tilde{r_2}, ..., \tilde{r}_{\tilde{N}})$. A number line is defined here as a half-open interval $[0, 1)$. This allows the definition of each accumulated probability interval at each iteration by its upper and lower limit. $\tilde{R}$ is treated as an arithmetic codeword $\hat{R}$, i.e., a real number $0 \leq \hat{R} < 1$:

$$\hat{R} \in [l(c), u(c)) \tag{10}$$

with $l(c)$ representing the lower limit at stage c of the encoding, and $u(c)$ the corresponding upper limit. The encoding then follows the principles of arithmetic decoding [12], [11], with the purpose of generating a constraint-adhering DNA sequence $B = (b_1, b_2, ..., b_O)$ from $\hat{R}$. The encoder is initialized as:

$$\begin{aligned} l(0) &= P_q(-1) = 0 \\ u(0) &= P_q(S-1) = 1 \end{aligned} \tag{11}$$

with S as the size of the source alphabet Σ. The sequence of symbol indices q_i is calculated in the following way:

$$q_i = \{q : l(i) \leq \hat{R} < u(i)\}, i = 1, 2, ..., O \tag{12}$$

where the upper and lower limits are updated for each value of i:

$$\begin{aligned} l(i) &= l(i-1) + (u(i-1) - l(i-1))P_q(q_i - 1) \\ u(i) &= l(i-1) + (u(i-1) - l(i-1))P_q(q_i) \end{aligned} \tag{13}$$

After each iteration, the symbol where $\{q_i = k\} \leftarrow \{m_k\}$ is appended to the sequence B. After reaching encoder stage O and the output of encoded symbol b_O, the encoded, constraint-adhering sequence B is returned.

Assuming that a codebook without constraints is used, i.e., the probability for each base to occur at each position is 25 %, then the output DNA sequence has a length of the number of bytes of the input sequence of the inner code, plus the number of CRC bytes, times 8 divided by 2, with an implementation-specific additional overhead of a single base that is required to safely flush the buffer at the end of the stream:

$$O = \frac{\tilde{N} \cdot 8}{2} + 1 \tag{14}$$

The additional overhead that the inner encoded introduces to adhere to constraints is described in Section 2.4 of the main manuscript.

5.4 Inner decoding

At the beginning of the decoding of a channel output sequence $Y = (y_1, y_2, ..., y_I)$, with the goal of finding the most likely encoded sequence $\tilde{B}$, an empty stack Q is initialized. Furthermore, an arithmetic coding (AC) instance a is initialized at stage 0 of the decoding:

$$\begin{aligned} l(0) &= P(-1) = 0 \\ u(0) &= P_q(S-1) = 1 \end{aligned} \tag{15}$$

For each possible element of Σ, the starting AC instance is copied and one iteration of arithmetic-style encoding is carried out, given the candidate sequence $\tilde{B}$ that is at this stage of the decoding comprised of one base per AC instance, $\tilde{B} = \{\tilde{b}_1\}$:

$$\begin{aligned} l(1) &= P_q(q_1 - 1) \\ u(1) &= P_q(q_1) \end{aligned} \tag{16}$$

After decoding of the i-th base, the upper and lower limits are as follows:

$$\begin{aligned} l(i) &= l(i-1) + (u(i-1) - l(i-1))P_q(q_i - 1) \\ u(i) &= l(i-1) + (u(i-1) - l(i-1))P_q(q_i) \end{aligned} \tag{17}$$

The estimation metric is then calculated for y_1 and b_1, as described in the main manuscript:

$$\mu(y_1, \tilde{b}_1) = \begin{cases} \log_2(2(1-p_t)) - R & \text{if } y_1 = \tilde{b}_1 \\ \log_2(2p_t) - R & \text{if } y_1 \neq \tilde{b}_1 \end{cases} \tag{18}$$

For each of the instances, the evaluation index, i.e., the index of the channel output sequence Y, is incremented by one, leading to the metric evaluation comparing in the next iteration y_2 with $\tilde{b}_2$. Additionally, four more

instances of the starting AC instances are copied, one for each $m \in \Sigma$, where it is assumed, that a deletion has taken place. The estimation metric is then simplified to:

$$\mu(y_1, \tilde{b}_1) = \log_2(2p_t) - R \tag{19}$$

Since for this instance it is assumed that a deletion has taken place, the evaluation index will not be incremented, i.e., in the next iteration, y_1 is compared to $\tilde{b}_2$ during metric evaluation. Finally, if $\tilde{b}_1$ is the same base as y_2, one more initial AC instance is copied that assumes an insertion has taken place. The metric used in this case is as follows:

$$\mu(y_2, \tilde{b}_1) = \log_2(2p_t) - R \tag{20}$$

The evaluation index is then incremented by two, i.e., in the next iteration, $\tilde{b}_2$ is compared to y_3. This approach cannot detect two insertions in succession. In the case of two consecutive insertions, the sequence will not decode properly and the information it contains has to be reconstructed by the outer code. Each instance is added to Q, which is then sorted according to the metrics of the AC instances. In the next decoder iteration, the ξ instances with the best metric are further extended and evaluated as described above, with ξ being user-defined in the configuration file. When the amount of decoded bytes of an AC instance reach a multiple of the step-size parameter $\tau + 1$ (the last byte being the 8-Bit CRC checksum added by the encoder), before adding the AC instance to Q, a CRC check is performed:

$$\mathrm{CRC}(\tilde{b}_{\tilde{\delta}-\tau}, ..., \tilde{b}_{\tilde{\delta}}) \tag{21}$$

If the CRC check fails, the AC instance is discarded. Otherwise, the output bytes, without the CRC byte, are stored and the AC instance is inserted into Q. After the processing of the last input base y_i and the successful last CRC check, the decoded output bytes without the added CRC bytes are returned, together with the final path metric.

5.5 Outer decoding

The output of the inner decoder can be decoded using the Raptor fountain code [9, 10]. For each received packet, s_f and δ_f is extracted. With the known distribution $\Omega(x)$ and the pseudo random number generator initialized with s_f as the seed, the degree d_f can be reconstructed. The vector v_f can then be reconstructed similar to the encoding. With v_f, the (intermediate) symbols that were used to create the packet can be extracted. To reduce the linear combination of each encoded symbol, we can replace each occurrence of an intermediate symbol by its linear combination of the input symbols. This is done by using G with v_f such that we get $v'_f = (v'_{f1}, v'_{f2}, \dots, v'_{f(V)})$, a vector representing the input symbols included in δ_f. With this information, either belief propagation or Gaussian elimination can be used to reduce the received packets to the input symbols. For the belief propagation algorithm, we can visualize the process using a graph where each encoded symbol δ_f has an edge to a source symbol h_j if $v'_f j = 1$. Figure 9 shows a minimal example graph for a Raptor code with the intermediate symbols removed, mapping 4 source symbols to 5 output symbols. The algorithm continues

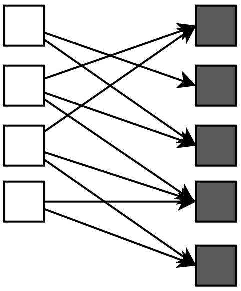

Figure 9: Graph representation of a Raptor code without intermediate symbols.

until there are no more symbols of degree 1 or if all input symbols were recovered. For each received packet with a degree of 1, the unique source symbol can be recovered, added to all connected output symbols and the involved edges can be removed [9]. While the belief propagation approach is considerably faster, it will only yield results if there are encoded symbols of degree 1 and (on the average) one additional symbol will be reduced to degree 1. Therefore, Gaussian elimination can be used as a backup to retrieve the source symbols as long as the matrix A is of rank k. For $A = (v'_1, v'_2, \dots, v'_r)^\top$ with $N \le r$ and $b = (\delta_1, \delta_2, \dots, \delta_r)^\top$ we can solve $Ax = b$ using Gaussian elimination with partial pivoting. If the solved matrix A' is equal to an identity matrix (or the first k rows for an over-determined system: $A'_{n,*} = I_n$), then b' contains the source symbols. Since the rows are filled in the order of highest inner-code metric first, the first k rows consist of the k inner-code output sequences

with the best metric. If the source symbols include a header chunk, the meta-information can be extracted, the checksum verified, the padding removed, and the file can be saved with the correct filename. Equation 22 shows how Gaussian elimination can be used to solve the minimal example of Figure 9 (with arbitrary data as b).

$$\begin{pmatrix} 0 & 1 & 1 & 0 \\ 1 & 0 & 0 & 0 \\ 1 & 1 & 0 & 0 \\ 0 & 1 & 1 & 1 \\ 1 & 0 & 1 & 1 \end{pmatrix} x = \begin{pmatrix} 011010110001\ldots \\ 101010101100\ldots \\ 111011010001\ldots \\ 001010111011\ldots \\ 110001101010\ldots \end{pmatrix} = b \xrightarrow{\text{gauss}} \begin{pmatrix} \mathbf{I_n} \\ \ldots \end{pmatrix} x = \begin{pmatrix} 101010101100\ldots \\ 010001111101\ldots \\ 001011001100\ldots \\ 010000001010\ldots \\ \ldots \end{pmatrix} = b' \tag{22}$$

References

[1] Grass, R. N., Heckel, R., Puddu, M., Paunescu, D. & Stark, W. J. Robust chemical preservation of digital information on DNA in silica with error-correcting codes. *Angewandte Chemie International Edition* **54**, 2552–2555 (2015).

[2] Press, W. H., Hawkins, J. A., Jones, S. K., Schaub, J. M. & Finkelstein, I. J. HEDGES error-correcting code for DNA storage corrects indels and allows sequence constraints. *Proceedings of the National Academy of Sciences* **117**, 18489–18496 (2020).

[3] Erlich, Y. & Zielinski, D. DNA fountain enables a robust and efficient storage architecture. *Science* **355**, 950–954 (2017).

[4] Löchel, H. F., Welzel, M., Hattab, G., Hauschild, A.-C. & Heider, D. Fractal construction of constrained code words for DNA storage systems. *Nucleic Acids Research* **50** (2021).

[5] Löchel, H. F., Eger, D., Sperlea, T. & Heider, D. Deep learning on chaos game representation for proteins. *Bioinformatics* **36**, 272–279 (2020).

[6] Löchel, H. F. & Heider, D. Chaos game representation and its applications in bioinformatics. *Computational and Structural Biotechnology Journal* **19**, 6263–6271 (2021).

[7] Organick, L. *et al.* Random access in large-scale DNA data storage. *Nature Biotechnology* **36**, 242–248 (2018).

[8] Welzel, M. *et al.* Natrix: a snakemake-based workflow for processing, clustering, and taxonomically assigning amplicon sequencing reads. *BMC Bioinformatics* **21** (2020).

[9] Shokrollahi, A. Raptor codes. *IEEE Transactions on Information Theory* **52**, 2551–2567 (2006).

[10] Luby, M., Shokrollahi, A., Watson, M. & Stockhammer, T. Raptor forward error correction scheme for object delivery. Tech. Rep. (2007).

[11] Bull, D. R. & Zhang, F. Lossless compression methods. In *Intelligent Image and Video Compression*, 225–270 (Elsevier, 2021).

[12] Witten, I. H., Neal, R. M. & Cleary, J. G. Arithmetic coding for data compression. *Communications of the ACM* **30**, 520–540 (1987).
